# *Clostridium difficile*: New Insights into the Evolution of the Pathogenicity Locus

**DOI:** 10.1038/srep15023

**Published:** 2015-10-08

**Authors:** Marc Monot, Catherine Eckert, Astrid Lemire, Audrey Hamiot, Thomas Dubois, Carine Tessier, Bruno Dumoulard, Benjamin Hamel, Amandine Petit, Valérie Lalande, Laurence Ma, Christiane Bouchier, Frédéric Barbut, Bruno Dupuy

**Affiliations:** 1 Laboratoire Pathogenèse des Bactéries Anaérobies, Institut Pasteur, Paris, France; 2 Plate-forme Génomique, Institut Pasteur, Paris, France; 3 AP-HP, Hôpital Saint Antoine National Reference Laboratory for C. difficile, Paris, France; 4 UPMC Univ Paris 06, GRC n°2, Epidiff, Paris, France; 5 CH, Hôpital Cambrai, Cambrai, France; 6 Hôpital de Villefranche-sur-Saône, Villefranche-sur-Saône, France

## Abstract

The major virulence factors of *Clostridium difficile* are toxins A and B. These toxins are encoded by *tcdA* and *tcdB* genes, which form a pathogenicity locus (PaLoc) together with three additional genes that have been implicated in regulation (*tcdR* and *tcdC*) and secretion (*tcdE*). To date, the PaLoc has always been found in the same location and is replaced in non-toxigenic strains by a highly conserved 75/115 bp non-coding region. Here, we show new types of *C. difficile* pathogenicity loci through the genome analysis of three atypical clinical strains and describe for the first time a variant strain producing only toxin A (A^+^B^−^). Importantly, we found that the PaLoc integration sites of these three strains are located in the genome far from the usual single known PaLoc integration site. These findings allowed us to propose a new model of PaLoc evolution in which two “Mono-Toxin PaLoc” sites are merged to generate a single “Bi-Toxin PaLoc”.

The Gram-positive spore-forming bacterium *Clostridium difficile* is the major etiological agent of intestinal diseases associated with antibiotic therapy, with clinical manifestations that range from diarrhoea to pseudomembranous colitis and possible death^1^. The incidence and severity of *C. difficile* infection (CDI) have significantly increased over the past fifteen years, mainly due to the emergence of new strain variants, such as hypervirulent PCR-ribotype 027 strains^1^. Therefore, CDI has a considerable impact on healthcare systems in North American and European hospitals^2^. Moreover, 23% of *C. difficile* infections are potentially undiagnosed due to the absence of clinical suspicion and suboptimum laboratory diagnostic methods^3^.

The major virulence factors of *C. difficile*, toxin A (TcdA) and toxin B (TcdB), are two members of the Large Clostridial Toxin (LCT) family, which are potent monoglycosyltransferases that disrupt the gut epithelium. Genes encoding TcdA (*tcdA*) and TcdB (*tcdB*) are located within the pathogenicity locus (PaLoc), a 19.6 kb chromosomal region that also contains three additional genes (*tcdR, tcdE* and *tcdC*). *tcdR* encodes an RNA polymerase sigma factor that positively regulates toxin expression^4^, *tcdE* encodes a bacteriophage holin required for toxin secretion^5^, and *tcdC* encodes a negative regulator of TcdR^6^. The PaLoc is always found in the same genomic location and is replaced in the non-toxigenic strains by a highly conserved 115/75 bp non-coding region^7^,^8^. A third unrelated binary toxin (CDT) is found in 23% of *C. difficile* strains, but its role in disease remains unclear^9^. This toxin is encoded in a separate region of the chromosome (CdtLoc) containing genes for both components of CDT (*cdtA* and *cdtB*) and a regulatory gene (*cdtR*)^10^.

The genetic polymorphisms of the PaLoc have been confined to a toxinotyping scheme, a PCR-RFLP-based method that was developed by Rupnik and colleagues^11^ that groups strains with identical changes in the PaLoc when compared to the other strains. In both Gram-negative and Gram-positive bacteria, many genes encoding virulence factors, including toxins, are located within mobile genetic elements, such as pathogenicity islands (PAIs). Unlike the PaLoc, PAIs contain direct repeats and insertion sequences, which are responsible for high-frequency deletions, duplications or amplifications, leading to a high level of variation and evolutionary diversities for virulence-factor-encoding genes. Even if *C. difficile* PaLoc does not fit the generally accepted definition of a PAI^12^, horizontal toxin gene transfer and PaLoc recombination events are the main mechanisms of toxin diversity^13^.

Comparative phylogenomics of well-characterised isolates of *C. difficile* revealed that the *C. difficile* population structure is divided into six distinct phylogenetic clades (Clades 1, 2, 3, 4, 5 and C-1)^8^,^14^. With the exception of Clade C-1, most of these clades include toxinogenic strains (A^+^B^+^or A^−^B^+^)^8^, which are mainly found in Clade 1 and to a lesser extent in Clades 2 and 3. Recently, toxinogenic strains were discovered in Clade 5^15^,^16^. The number of toxinogenic genotypes that have been identified across each clade varies widely^8^, which might be consistent with independent PaLoc acquisition followed by clonal expansion. Thus, the relationship between PaLoc types and *C. difficile* strains is likely in constant evolution, and recent PaLoc acquisitions and exchanges likely play an important role in the under-diagnosis of CDI.

In this work, we show a new type of genomic organisation of the *C. difficile* PaLoc through the analysis of three atypical strains isolated from CDI. We describe for the first time a variant strain producing only TcdA (A^+^B^−^) and new toxigenic strains (A^−^B^+^CDT^+^) strains that belong to Clade C-I. For the latter, we found that both PaLoc and CdtLoc are located in the same genomic region. Importantly, the PaLoc can be located at different sites of the genome, distant from the single, yet known, PaLoc integration site, thereby opening new questions regarding PaLoc evolution. Based on the sequence analysis of these new PaLoc variants, we discuss a model merging two “Mono-Toxin PaLoc” to generate a single “Bi-Toxin PaLoc”.

## Materials & Methods

### Bacterial strain identification

The RA09-070 strain was isolated during a French national prospective and multicentric study of CDI^17^, and the SA10-050 and CD10-165 strains were sent to the National Reference Laboratory for *C. difficile* for characterisation (Paris, France). The identification of the three strains as *C. difficile* was confirmed using Matrix-assisted laser desorption ionisation (Maldi) time-of-flight (Tof) mass spectrometry (Brucker) and the glutamate dehydrogenase (GDH) component of the C. diff Quik Chek Complete assay (Alere, Jouy-en-Josas, France). DNA was extracted with the InstaGene Matrix kit (Bio-Rad Laboratories, Hercules, California, USA). The entire PaLoc was explored by the amplification of fragments of both *tcdA* (A1, A2 and A3) and *tcdB* (B1, B2 and B3) as described in the toxinotyping schema that was developed by Rupnik *et al.*^11^. PCR amplifications of the *tcdC*, *cdtA* and *cdtB* genes were performed using primers described elsewhere^11^,^17^. PCR-ribotyping was performed as recommended by Bidet *et al.* and capillary-gel based electrophoresis patterns were compared to a collection of 26 well-defined ribotypes (001, 002, 003, 005, 012, 014/020/077, 015, 017, 018, 019, 023, 027, 029, 046, 050, 053, 056, 070, 075, 078, 126, 081, 087, 106, 117 and 131)^18^. The strains were characterised by MLST and ST types were identified using two MLST schemes that were developed by Lemee *et al.*^19^ and by Griffiths *et al.*^20^.

*In vitro* toxin B production was tested by the cytotoxicity assay on MRC-5 cell monolayer as described elsewhere^21^. Toxin detection was also tested by the C. diff Quik Chek Complete assay (Alère, Jouy-en-josas, France) and Vidas *C. difficile* Toxin A and B (Biomérieux, Marcy l′Etoile, France), performed directly on colonies. Molecular methods, such as *illumigene C. difficile* (Meridian Bioscience, OH, USA), Xpert *C. difficile* (Cepheid, CA, USA), Rida Gene *C. difficile* and Toxin A/B (R-biopharm, Darmstadt, Germany), were performed from colonies, as recommended by the manufacturers.

### DNA sequencing and genome assembly

DNA was extracted from overnight culture growth in TY media, inoculated from one colony as previously described^22^. Single-end multiplex libraries were created, and the sequencing was performed using the Illumina HiSeq 2000 platform. The read length was 110 bp, and isolates were sequenced at least to an average coverage of 100-fold (20, 5 and 6 billion reads, respectively, for RA09-70, SA10-050 and CD10-165). Sequencing reads were first scanned to remove the adaptor sequences and were then assembled *de novo* into contigs using Velvet^23^. Thereafter, contigs were reorganised using Blast alignment against the genome sequence of the reference *C. difficile* 630 strain. All of the contigs that did not match the sequence of the reference strain were localised at the end of the contigs that were assembled to obtain a whole genome scaffold for each of the isolates. Finally, the Microscope work flow^24^ was used for an automatic functional annotation of each CDS.

The sequenced reads of the *C. difficile* RA09-70, SA10-050 and CD10–165 strains were deposited in the Sequence Read Archive (Accession Numbers PRJNA255280, PRJNA260039 and PRJNA260040) and their annotated genome in the DDBJ/EMBL/GenBank databases (GenBank Accession Numbers JPPA00000000, JRHM00000000 and JRHN00000000).

### Phylogenetic Analyses

Strains representative of the diversity of *C. difficile* PCR-ribotypes and of the six clades (1, 2, 3, 4, 5 and C-I) were chosen (Table S1). The maximum likelihood trees with 500 bootstrap replicates were constructed by performing a phylogenetic analysis of the *cdu1* and *ccd3* (Fig. S3) genes and of the TcdA and TcdB proteins (Fig. S8 and Fig. S9) using MEGA 6.0^25^.

### Analysis of the *tcdR* regulatory region

We performed an *in silico* analysis of the regulators (CodY and CcpA) and sigma factors (TcdR and SigD) binding sites that were present in the *tcdR* promoter regions of strains CD630, CD10-165/SA10-050 and RA09-70. For this analysis, we scanned the 400 bp intergenic region upstream of *tcdR* gene with the published consensus binding sites of CodY “AATTTTCWGAAAATT”^26^, CcpA “RRGAAAANGTTTTCWW”^27^ and the -35 box of the TcdR promoter “TTTACA”^28^ and the SigD promoter “TAAAN(13–19)CGW”^29^. We adjusted the number of mismatches in each consensus to recover the experimentally verified *tcdR* binding sites of CD630 for CodY (three known sites recovered with four mismatches), CcpA (2 known sites recovered with three mismatches), TcdR (2 known sites recovered with 0 mismatches) and SigD (1 known site recovered with 0 mismatches) (Fig. S6).

### UviB holin activity assay

The pBRQ(ΔRBS), a derivative of the pJN4^30^ deleted in the Sλ gene ribosomal binding site (RBS), was used to create constructs in which the UviB gene was placed under the control of the λ pR’ promoter (late transcription regulatory (LTR) region, spanning from the 3′ end of the antiterminator Q gene to the first base pair of the S holin gene)^30^. The *uviB* gene coding sequence, including its own ribosomal binding site, was PCR amplified, digested and ligated to pBRQ(ΔRBS). To test the holin activity of UviB, lysogens of *Escherichia coli* strain MC1061 for a defective λ prophage bearing a nonsense mutation in its holin gene (λCi_857_Sam7) or carrying a deletion in holin and endolysin genes [λCmrD(SR)] were used as hosts for the plasmid constructs^5^, including plasmids pJN5 and pRG32, which carry the genes encoding the λ holin S^105^ and TcdE, respectively^5^. Both λ (Sam7) and λCmrΔ(SR) encode a thermo-sensitive CI repressor (cI857) and are induced upon shifting the culture temperature from 30 °C to 37 °C^31^. Resultant strains were grown in LB broth at 30 °C until the OD600 reached 0.15–0.25 before the thermo-induction of the λ prophage at 42 °C for 15 min. Bacterial growth and lysis at 37 °C were then followed by monitoring the absorbance at 600 nm at 15 min intervals.

## Results

### Microbiological and molecular analysis of new variant *C. difficile* strains

The three strains that were investigated in this study were isolated from clinical samples. The RA09-070 strain was isolated from a 60-year-old man with antibiotic-associated diarrhoea, which was successfully treated by oral metronidazole without further recurrence. The SA10-050 strain was isolated from an 84-year-old man who presented febrile diarrhoea during the course of treatment for multiple myeloma. CDI was successfully treated by oral metronidazole. Finally, the CD10-165 strain was isolated from a 74-year-old man who presented abundant diarrhoea and severe pseudomembranous colitis. The patient deceased in the context of multiple organ failure and shock, and the death was directly attributed to *C. difficile*. The complete case report of these strains is described in the supplementary data.

Surprisingly, the immuno-enzymatic tests, the cytotoxicity assay and molecular tests that were used for the diagnosis of these *C. difficile* strains displayed insufficient results. Toxin detection of the RA09-070 strain was positive with the Vidas and with the C. diff Quik Chek Complete immunoassays (weak band) (Table 1A). However, this result was negative using the MRC-5 cell cytotoxicity assay, which detects TcdB only, suggesting that the RA09-070 strain produced TcdA but not TcdB. This results was consistent with the detection of TcdA by a dot blot experiment performed from the crude extract of the strain (Fig. S1). This result was partially confirmed by commercially available molecular diagnostic tests. Indeed, the detection of toxin genes was negative when using Xpert *C. difficile* (a diagnostic assay that targets only *tcdB*) and positive when using the Rida Gene *Clostridium difficile* Toxin A/B (a PCR-based method simultaneously detecting *tcdA* and *tcdB*) but was unexpectedly negative with *illumigene C. difficile*, which targets a highly conserved region of *tcdA* (Table 1A).

The toxin detection assay of the SA10-050 and CD10-165 strains also presented insufficient results: detection was negative with the Vidas immunoassay but positive with C. diff Quik Chek Complete and the MRC-5 cell cytotoxicity assay (Table 1A), suggesting that both strains produced only TcdB. Consistently, we were not able to detect TcdA from the crude extracts of both strains (Fig. S1). This result was also partially confirmed by molecular analysis. As expected, the detection of toxin genes of both strains was negative using *illumigene C. difficile* and positive with Xpert *C. difficile* (Table 1A). However, toxin gene detection was surprisingly negative with the Rida Gene *Clostridium difficile* Toxin A/B.

In addition, we showed all of the strains were negative for binary toxin, whatever the method used (Xpert *C. difficile* or home-made PCR) and for the PCR amplification of the *tcdC* gene (Table 1A).

The results of the toxinotyping of the three strains were also unexpected. PCR for B1, B2, B3 and A3 were negative for the RA09-070 strain, whereas PCR fragments A1 and A2 of the *tcdA* gene were positive. For the SA10-050 and CD10-165 strains, all three PCRs covering *tcdA* (fragments A1, A2, A3) were negative, and only the B1 and B2 fragments covering the *tcdB* gene were positive. These results suggest that these strains belong to new toxinotypes.

**Figure 1:**
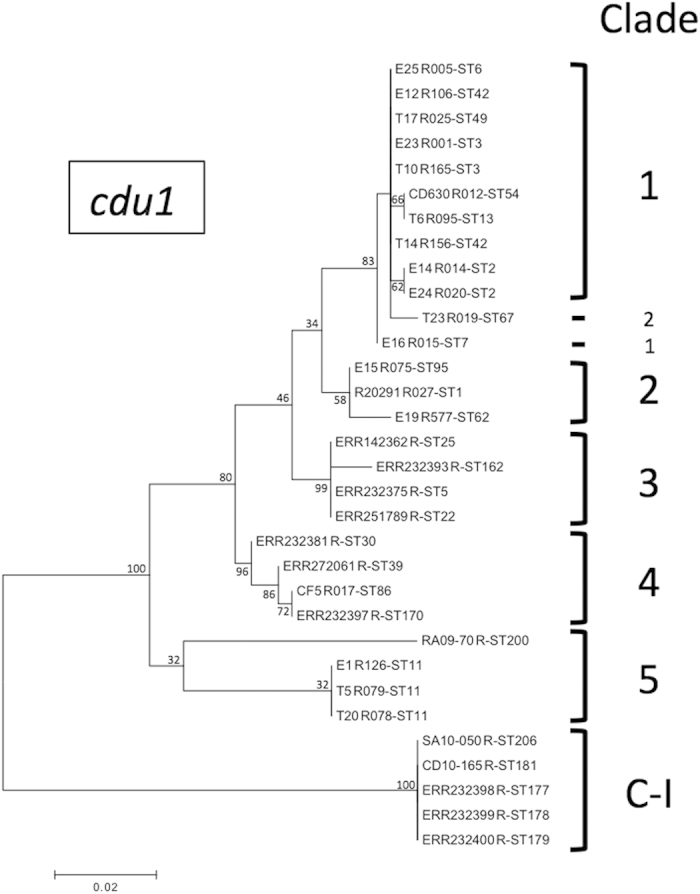
Phylogenic relationship of the *cdu1* genes. Maximum likelihood tree reflecting the similarity of *cdu1* gene from representative clade *C. difficile* strains.

The PCR-ribotype profiles of the strains (Fig. S1) were unusual, *i.e.,* they did not match any of the most frequent PCR ribotypes^17^. Moreover, *in silico* MLST analysis (Table 1B) showed that the RA09-70 strain was close to strains that belong to the sequence type ST63, as reported by Lemee *et al.*^19^, and to ST200, as reported by Griffiths *et al.*^20^. The SA10-050 and CD10-165 strains did not correspond to any sequence type that was reported by Lemée *et al.* but are respectively close to strains belonging to the ST206 and ST181 types that were reported by Griffiths *et al.* (Table 1B). By generating maximum likelihood trees from the *cdu1* and *cdd3* genes of at least three *C. difficile* strains per clade (Fig. 1 and Fig. S3), we showed that SA10-050 and CD10-165 belongs to Clade C-I. RA09-70 is a member of Clade 5 based on *cdu1* tree (Fig. 1) but apparently belongs to a distinct group according to the *cdd3* tree (Fig. S3). More strains as RA09-70 are needed to confirm its Clade.

In conclusion, we have characterised three clinical strains of *C. difficile* belonging to new toxinotypes displaying very specific and atypical features: the RA09-70 strain is the first description of a variant strain harbouring only the toxin-A-encoding gene. The SA10-050 and CD10-165 strains form, along with the recently described strains of toxinotypes XXX and XXXI^16^,^32^,^33^, a new group of A^−^B^+^CDT^+^ variants lacking a complete *tcdA* gene.

### Atypical Genetic Organisation of the RA09-70, SA10-050 and CD10-165 PaLoc

**Figure 2:**
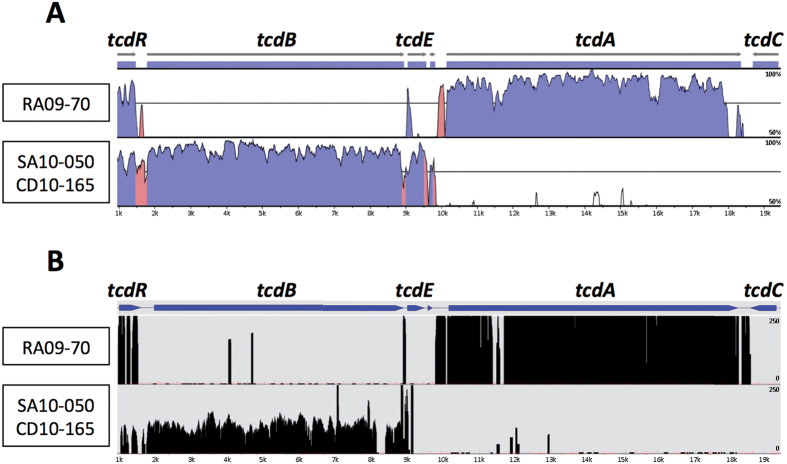
Genetic organization of the RA09-70 and SA10-050/CD10-165 PaLoc. (**A**) PaLoc of the 3 clinical strains were aligned with the CD630 PaLoc using LAGAN^48^ (http://lagan.stanford.edu) and visualized by VISTA^49^. The X-axis represents the CD630 PaLoc and the Y-axis the percent identity (from 50 to 100%) of the compared strain PaLoc by windows of 100 bp. The pink regions correspond to conserved non-coding sequences and the dark blue regions to CDS. (**B**) Mapping of the sequencing reads on the CD630 PaLoc. Alignment of sequencing reads onto the CD630 PaLoc was made using Blast^50^ and visualized using COV2HTML^51^. The X-axis represent the CD630 PaLoc and the Y-axis the mapping coverage of the sequence reads (from 0 to 250).

To resolve the discrepancies between the toxin assay and the results of the molecular diagnostic analysis, we performed a whole-genome-sequencing approach for each strain to evaluate changes in the PaLoc genes that could explain the absence of toxin gene detection by some of the molecular *C. difficile* tests^16^. A *de novo* genome assembly showed that the PaLoc of RA09-70 strain is restricted to *tcdR* and *tcdA* genes (Fig. 2A). To ensure that the *tcdB*, *tcdE* and *tcdC* genes were really absent rather than lost in the assembly process, a deep search in the raw-data sequences was performed. By mapping all of the sequence reads onto the classic PaLoc sequence of the CD630 strain (Fig. 2B), we did not detect sequences from the raw-data file hybridising with the classic PaLoc sequence and thereby confirmed that *tcdB*, *tcdE* and *tcdC* genes were not present in strain RA09-70, not even in truncated form. However, we identified a new, putative CDS of 216 bp between the *tcdR* and *tcdA* genes. This CDS encodes a protein of 71 amino acids containing a complete domain (IPR024405) related to the prophage protein BhlA/UviB, which are potentially involved in endolysin and bacteriocin secretion, respectively^34^,^35^.

In the SA10-050 and CD10-165 strains, *tcdR*, *tcdB* and *tcdE* were the only genes present within the PaLoc (Fig. 2A). Using the aforementioned mapping method (Fig. 2B), we confirmed that *tcdA* and *tcdC* were completely absent. Interestingly, next to *tcdE,* we found a gene encoding an endolysin protein (CwlH) with N-acetylmuramoyl-L-alanine amidase activity, which supports the phage origin of the PaLoc and is consistent with the holin activity of TcdE^5^. Indeed, bacteriophage holins form holes in the host cell membrane to allow prophage-endolysin to cross the membrane and degrade bacterial peptidoglycan, resulting in cell lysis and the release of phage particles^36^.

In addition, to clarify the structure of the PaLoc of these three strains, whole genome sequencing confirmed the integrity of the *tcdA* gene for the RA09-070 strain and the *tcdB* gene for the SA10-050 and CD10-165 strains. Moreover, the complete genome sequencing of the three strains revealed an unusual amino acid sequence variability of *tcdA* and *tcdB* (83% and 89% identity, respectively) compared to strain CD630 (Fig. S8 and Fig. S9). The weak homology between the sequences of toxin genes and the primers that were used for the A3 and B3 PCR fragments could explain the absence of PCR fragment amplification during toxinotyping as well as the detection of toxin genes with *illumigene C. difficile* and Rida Gene *Clostridium difficile* Toxin A/B tests, although the genomic targets of these assays are not publicly reported (Table 1A). Finally, the negative results that were obtained with the immunoassays may also be due to changes in the amino acid sequence of the toxins, thereby preventing recognition by the monoclonal antibodies that are used in the commercially available assays.

Interestingly, the whole genome sequencing approach identified a complete *cdt* locus in both the SA10-050 and CD10-165 strains that was not detected by molecular methods (Table 1A). This result seems to be due to the absence of sequence homology between the binary toxin genes of the SA10-050 and CD10-165 strains and the primers that were used for their amplification^11^ (Table 1A).

### PaLoc localisation of the RA09-70, SA10-050 and CD10-165 strains

Despite thousands of sequenced *C. difficile* strains from transmission or evolutionary studies^8^,^37^,^38^, the *C. difficile* PaLoc was always found in the same genomic position that was replaced in non-toxinogenic strains by a highly conserved 115/75 bp non-coding region^7^,^8^. However, the recent description of a new toxinotype strain^16^ suggested that PaLoc may also be inserted at other genomic sites, although the authors could not indicate the exact position and boundaries regions of the corresponding PaLoc^16^. Interestingly, in the three atypical strains, the usual classical PaLoc integration site contained the 75 bp PaLoc-replacing sequence and five genes that were previously described in non-toxigenic strains of Clade 5 (for the RA09-70 strain) and Clade C-I (for the SA10-050 and CD10-165 strains)^8^, whereas the alternative PaLoc was present elsewhere in the genome.

**Figure 3:**
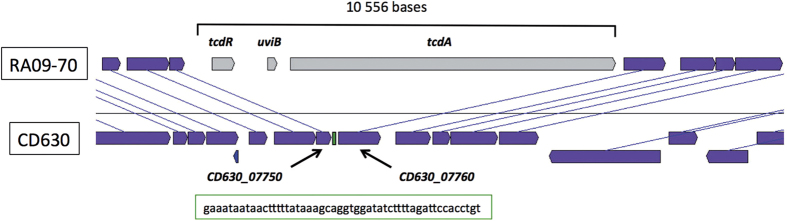
Localization of the RA09-70 PaLoc insertion site. The RA09-70 PaLoc is inserted between CD630_07750 and CD630_07760 genes of CD630. The RA09-70 PaLoc size is 10 556 bases and the 51 bases of the intergenic region lost in the RA09-70 strain are boxed in green. The syntheny was done using MaGe in the Microscope platform^24^, CDS considered in syntheny are purple.

**Figure 4:**
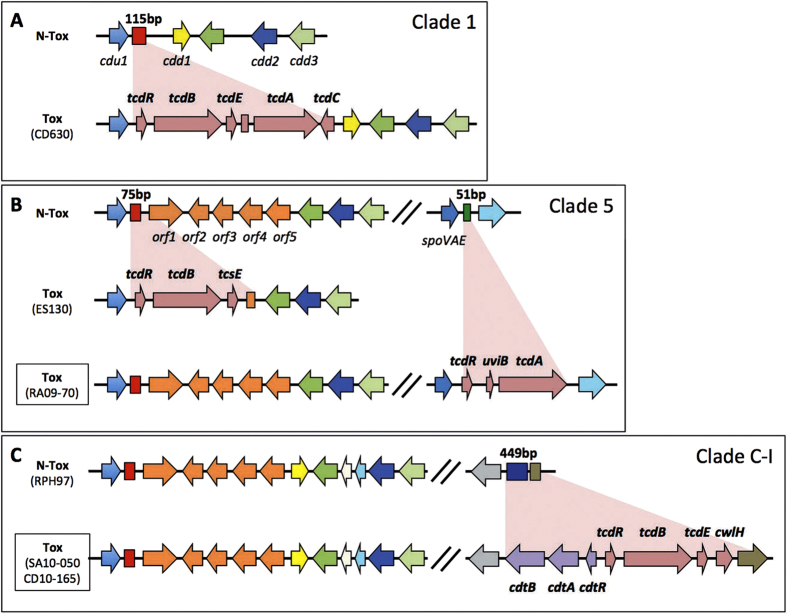
PaLoc organization of the RA09-70 and SA10-050/CD10-165 strains. Schematic description of the single PaLoc insertion site between non-toxigenic (N-Tox) and toxinogenic (Tox) isolates representing Clade 1 (**A**), 5 (**B**) and C-I (**C**). PaLoc insertion site of the RA09-70 and SA10-050/CD10-165 strains correspond respectively to the Clade 5 and C-I. Colored boxes represent PaLoc replacing sequences (red: 115/75 bp, green: 51 bp) and the five genes (orf1-5) identified in this location in Clade 5 and C-I^8^ are represented by orange arrows. Strain ES130, described by Elliot and Coll.^15^ belongs to Clade 5.

According to whole-genome data, the RA09-70 PaLoc size (10.5 kb) is smaller than the usual PaLoc size (19.6 kb) of most of toxinogenic strains. Surprisingly, we found that genes flanking the PaLoc of the RA09-70 strain have no similarity with the *cdu1* and *cdd1* genes upstream and downstream, respectively, of the classical studied PaLoc integration site to date^8^. However, these genes have more than 99% sequence identity with CD630_07750 and CD630_07760 genes (Fig. 3). Our analysis shows that both genes are conserved in RA09-70 and that 51 bases of the intergenic region were lost compared to the same region in the CD630 strain. We noted that there is no homology between this 51 base replacement sequence in the RA09-70 strain and the 115/75 bases of the classical PaLoc insertion sequence (Fig. 4b). As mentioned above, the PaLoc of the SA10-050 and CD10-165 strains is also inserted at a different genomic location, although it is more difficult to precisely identify the PaLoc insertion site. Indeed the PaLoc boundary regions in strains SA10-050 and CD10-165 are not conserved in any other available genome of *C. difficile*. Therefore, we assembled *de novo* the raw data reads into scaffold genomes from five Clade C-I strains that were described in Dingle *et al.*^8^ (Table S1). From the genome analysis of the RPH97 strain, we found the boundary regions of the CdtLoc-PaLoc region of the SA10-050 and CD10-165 strains and precisely determined the integration position (Fig. 4c) with a replacement of 449 bases compared to the RPH97 strain. This C-I replacement sequence has no homology with that of Clades 1 and 5.

In addition, the PaLoc of both strains is present next to the complete *cdt* locus (Fig. 4c), which has never been described before. The atypical genomic location of the RA09-70 PaLoc compared to the CD630 PaLoc and the organisation of PaLoc—CdtLoc of both SA10-050 and CD10-165 strains were confirmed by PCR experiments using internal and flanking PCR primers (Fig. S2).

To our knowledge, this is the first study clearly demonstrating that the PaLoc of *C. difficile* can be inserted at different genomic locations distant from the usual, unique PaLoc integration site that has been considered to date.

### Sequence diversity of the RA09-70, SA10-050 and CD10-165 PaLoc and CdtLoc

As previously mentioned, *C. difficile* strains are distributed as variant toxinotypes based on the changes in their PaLoc, and to date, 32 such groups have been defined^11^. In contrast to the PaLoc, for which several truncated versions are known, few variations of the CdtLoc (*cdtR*, *cdtA* and *cdtB*) have been reported. CdtLoc is present either as a whole- or a single-truncated version and is replaced by a 68 bp sequence in the chromosomal location of the *cdt-*negative strains^10^. By analysing the *tcdR* and *cdtR* sequences, we assessed the variability of PaLoc and CdtLoc to study the genetic relatedness of the RA09-70, SA10-050 and CD10-165 strains among the *C. difficile* strains, as previously detailed^39^.

When we searched for the homology of the TcdR and CdtR proteins between the reference strain CD630 and their homologs in a wide range of PCR-ribotype strains, we found that both regulators were highly conserved, with sequence identities of >97% and >95%, respectively (Table 2 and Table S1). In contrast, TcdR of the RA09-70 and SA10-050/CD10-165 strains shared only 73% and 75%, respectively, amino acid identities with TcdR of all of the other PCR-ribotype strains that were selected in this study, including the reference CD630 strain (Table 2 and Table S1). Moreover, the amino acid identity of CdtR present in the SA10-050 and CD10-165 strains varies from 59% to 62% compared to CdtR of all of the studied CDT-positive strains (Table 2 and Table S1). Thus, the low identity of the TcdR and CdtR regulators between the RA09-70, SA10-050 and CD10-165 strains and the representative strains of the main PCR-ribotypes suggests that sequences of their PaLoc and CdtLoc must be distinct enough to consider a long-term divergence compared to those of the *C. difficile* strains that have been studied to date.

### The holin-like protein BhlA/UviB: a substitute of TcdE?

Based on the analysis of more than 1000 sequenced genomes of toxigenic *C. difficile* strains^8^,^22^, we observed that a holin-encoding gene is always present and corresponds to the class I holin TcdE^5^ for the majority of *C. difficile* strains. Recently, Elliot and colleagues^15^ showed in the *C. difficile* ES130 strain (Clade 5) that *tcdE* was replaced in the PaLoc by the *tcsE* gene, encoding a holin-like protein that is potentially required for secretion of *Clostridium sordellii* cytotoxin TcsL^8^,^40^. In the RA09-70 strain, we found that *tcdE* is substituted by a gene encoding a protein that contains a full-length domain homologous to bacteriophage holin protein BhlA/UviB. This putative holin protein of 71 amino acids has a single N-terminal transmembrane domain (TMD) and several positively charged amino acids in the C-terminus, which are structural features of class III holins (Fig. S4). Surprisingly, this protein shares no significant homology with TcdE but has 34% and 31% amino acid identities with UviB of *C. perfringens* and BhlA of *B. subtilis*, respectively. UviB was assumed to play a role in bacteriocin secretion^41^, whereas BhlA, which was characterised as a new type of holin-like protein^42^, is likely required for the secretion of the BlyA, an N-acetylmuramoyl-L-alanine amidase that is associated with SPβ-phage-mediated cell lysis^35^. To annotate the putative holin-like protein of RA09-70, we constructed a phylogenetic tree by comparing the RA09-70 protein with both BhlA homologues in *Bacillus* species (*B. subtilis, B. laterosporus, B. licheniformis* and *B. pumilus*) and UviB homologues in Clostridium species (*C. perfringens, C. botulinum, C. tetani and C. sporogenes*). We found that the RA09-70 protein belongs to the UviB subtree (Fig. S4) and therefore was annotated as UviB.

**Figure 5:**
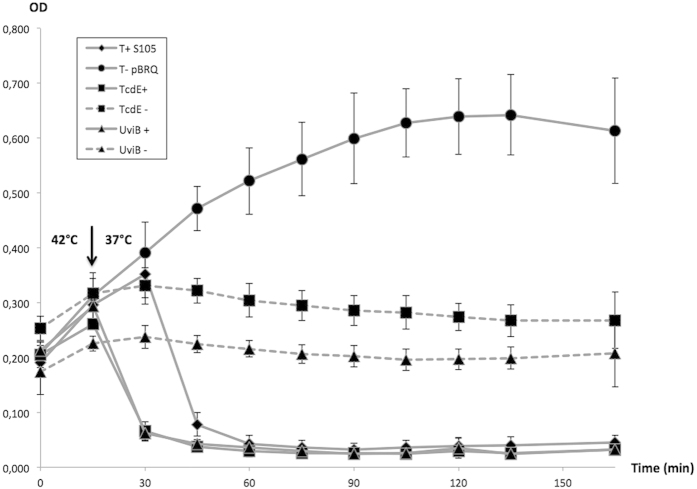
Testing holin fonction of UviB in *E. coli*. Lysis curves of *E. coli* λ lysogenic culture carrying λcI_857_Sam7 (full) or λCmrΔ(SR) (dotted) and plasmid expressing in trans S^105^ (T+), TcdE and UviB. *E. coli* λcI_857_Sam7 carrying pBRQ was used as negative control (T−). Curves are a compilation of 5 independent cultures (3 for UviB−).

Then, we sought to evaluate whether RA09-70 UviB has a holin-like activity by complementing an *E. coli* λ lysogen that is defective for the λ holin as previously performed for TcdE^5^. As previously mentioned, by forming holes in the host cell membrane, holin allows a prophage-encoded endolysin to cross the membrane and to attack the murein, resulting in cell lysis and in the release of phage particles^36^. To test the holin activity of RA09-70 UviB, we cloned the *uviB* gene in a heat-inducible expression plasmid that we used to complement *E. coli* λ lysogen (cI_857_Sam7), which has a functional endolysin gene but a nonsense mutation in its holin gene (Sam7)^5^. To confirm the functionality of this system, we expressed the λ holin (S^105^) in *E. coli* MC1063 λ (cI_857_Sam7) and observed that bacterial lysis is completed 45 minutes after heat induction (Fig. 5). In addition, no lysis was observed after the induction of the *E. coli* λ lysogen carrying the empty vector. When expressed in *E. coli* MC1063 λ (cI_857_Sam7), UviB induced complete bacterial lysis within fifteen minutes after heat induction, similarly to TcdE (Fig. 5). As for the λ holin (S^105^), lysis induced by the expression of UviB required the expression of the λ endolysin. In fact, the expression of UviB and TcdE in the *E. coli* λCmrΔ(SR) strain carrying a deletion in the holin and endolysin genes did not cause lysis (Fig. 5). These results indicate that the lysis of *E. coli* was not due to the over-expression of UviB, thereby demonstrating that UviB functions as a phage holin, similar to TcdE.

These results confirm that a holin-like gene is always present in the PaLoc in addition to the TcdR-encoding gene, as all known toxigenic strains that have been sequenced to date possess either TcdE or substitutes of TcdE, such as TscE^15^ or UviB (this study). These findings reinforce the importance of holin-like proteins in *C. difficile* toxin secretion^5^.

## Discussion

The under-diagnosis of *C. difficile* infections across Europe is due in part to the use of sub-optimal detection methods^3^ and possibly to the emergence of new variant strains that cannot be detected by the diagnostic tools that are available in different laboratories. We demonstrated in this work through the characterisation of new *C. difficile* variants that standard diagnostic assays may be impeded by changes in the PaLoc and CdtLoc. This event may result from major sequence variations in toxin genes that do not match the primers that are used in certain molecular methods or from the complete deletion of *tcdA*^15^,^16^ or *tcdB*. In the present study, we describe a clinical isolate A^+^B^−^, a finding that to the best of our knowledge has never been reported before for the many *C. difficile* strains that have studied. Importantly, we present here unique evidence that the PaLoc can be located at other sites of the genome than is usually the case for classic *C. difficile* strains.

The inconsistent results of the molecular and immunoassays that were performed in the three strains of this study perfectly illustrate that no single test could completely characterise all *C. difficile* isolates. Particularly, the absence of a diagnostic test for *cdt* genes leads to underestimate the presence of CdtLoc among toxinogenic strains. In contrast, a cell cytotoxicity assay was the only diagnostic test that consistently detected the cytopathic effect that was caused by the production of toxin B from strains SA10-050 and CD10-165. The presence of toxinogenic A^+^B^−^ strains, such as RA09-070, whose changes in amino acid sequence could limit the use of commercial immunoassays, would not even be diagnosed by cell cytotoxicity assays. Thus, our results underline the potential impact of target sequence modification in clinical strains on the results of routine diagnosis assays. The suboptimum diagnosis of such atypical strains may have direct implications for patients who will not received appropriate treatment and for who contact precautions to avoid nosocomial transmission will not be implemented. Although these new *C. difficile* variant strains seems to be very uncommon in clinical practice, our data suggest to use diagnostic tests that detect both toxins A and B or nucleic acid amplification assays targeting both *tcdA* and *tcdB* genes.

The whole genome sequence analysis of the RA09-70 and SA10-050/CD10-165 strains allowed us to demonstrate for the first time that the PaLoc can be located at different sites in the *C. difficile* genome. Moreover, we showed that elements inserted within the classical PaLoc integration site of these strains belong to different clades defined based on the flanking chromosomal sequences of the PaLoc^8^,^43^. We also show that insertion in the element of the RA09-70 strain is similar to the Clade 5 non-toxinogenic strains^8^ (Fig. 4b), which usually produce binary toxins^15^. However, although we clearly localised the PaLoc variant of this strain next to the *spoVAE* gene (CD630_07750) (Fig. 4b), we could not find genes of the CdtLoc. We deduced from this analysis that the PaLoc-replacing element of the SA10-050/CD10-165 strains belongs to Clade C-I (Fig. 4c), known to produce neither toxin A nor toxin B^8^. Accordingly, these strains seem to represent the first examples of a toxinogenic (A^−^B^+^) strain in this clade (Fig. 4c).

The identification of variable-sized PaLoc, integrated at different sites of the *C. difficile* genome, raised intriguing questions regarding PaLoc evolution, which seems to have been leading to the classical PaLoc observed in the majority of *C. difficile* strains.

**Figure 6:**
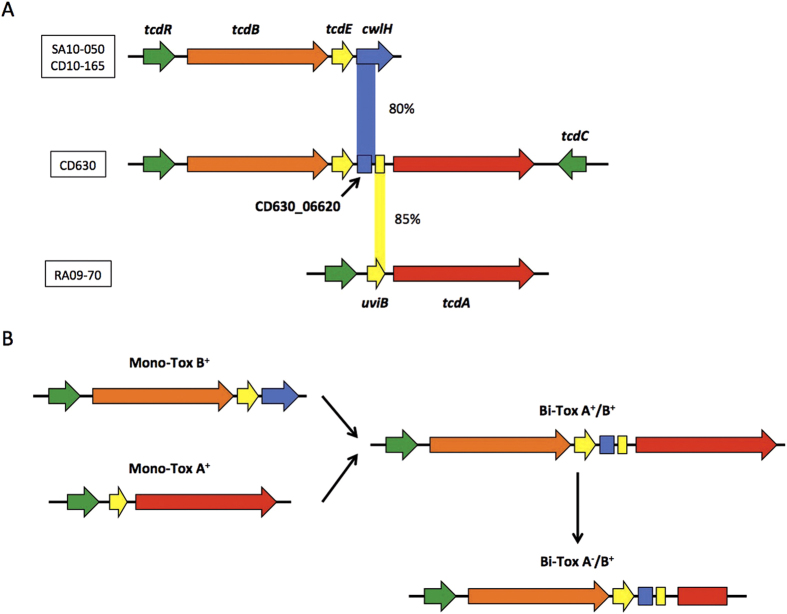
Model of the PaLoc Evolution from “Mono-Toxin Paloc” to “Bi-Toxin PaLoc”. Genes are identified by their color. (**A**) Fusion of two mono-toxin PaLoc sequence to form a single bi-toxin PaLoc. Nucleotide alignments of both SA10-050/CD10-165 *cwlH* gene and RA09-70 *uviB* gene to the intergenic region between *tcdE* and *tcdA* of CD630 are represented by colored column with their percent identity. (**B**) Model of (**C**). difficile PaLoc evolution.

Based on the obtained sequences of the PaLoc variants from the three strains studied here, we suggest that the classical PaLoc, *i.e.,* a “Bi-Toxin PaLoc”, might be the result of a fusion of two “Mono-Toxin PaLoc” from ancestral Clostridia strains through multiple independent PaLoc acquisitions, leading to the stabilisation of the “Bi-Toxin PaLoc”. Our hypothesis is supported by the sequence similarities that were observed between the *cwlH* and the *uviB* genes present within the PaLoc of the SA10-050/CD10-165 and the RA09-70 strains, respectively, and the intergenic region between *tcdE* and *tcdA* genes within the PaLoc of the reference strain CD630 (Fig. 6A). Indeed, the 5′ part of the endolysin encoding gene, *cwlH*, is highly similar (80% nucleotide identity) to the pseudogene CD630_06620 next to the *tcdE* gene of the CD630 PaLoc (Fig. 6A and Fig. S5). The complete match of CD630_06620 with the *cwlH gene* of SA10-050/CD10-165 strains confirmed its annotation as a phage-like endolysin fragment-encoding gene^44^. In addition, the C-terminal encoding part of *uviB* matches (85% nucleotide identity) a 136 bp intergenic region just upstream of the *tcdA* gene of the CD630 PaLoc (Fig. 6A and Fig. S5). This second match is localised immediately after the first match that was observed between the gene encoding *cwlH* and CD630_06620 (Fig. 6A). Hence, the two regions present side by side in the CD630 PaLoc that partly overlap the *cwlH* and the *uviB* from the PaLoc variants are very likely remnants of the fusion of two “Mono-toxin PaLoc” regions that gave rise to a single “Bi-Toxin PaLoc”. During this fusion, only one holin (TcdE) was conserved, and the endolysin (CwlH) seems to have been removed to avoid its potentially lethal effects on the membrane of bacteria^5^. Throughout the evolution of *C. difficile* strains, variations in the PaLoc could derive from the “Bi-Toxin PaLoc” by sequence changes or deletions of the toxin genes (Fig. 6B).

Furthermore, we also performed an *in silico* analysis of the *tcdR* promoter regions of the CD630, RA09-70 and CD10-165/SA10-050 strains. We found that the regulator and sigma factor binding site patterns of CD630 and CD10-165/SA10-050 strains were closer than the similarity with RA09-70 (Fig. S6). This result adds one more argument to our “fusion model” (Fig. 6), where the *tcdR* gene of the “Bi-toxin PaLoc” (CD630) comes from the “Mono-toxin B PaLoc” (CD10-165/SA10-050) rather than from the PaLoc of RA09-70. Such a PaLoc evolution model anticipates that diverse PaLoc forms had to be transferred among *C. difficile* strains, a finding that has recently been demonstrated experimentally for the CD630 PaLoc by a conjugation-like mechanism^45^. In this scenario, the *tcdC* gene, which is not present in the “Mono-toxin PaLoc” strains, must have been provided by another event because *tcdR* and *tcdC* genes have evolved divergently in the studied strains^39^.

The same model could be suggested for other pathogenic clostridia, such as *C. sordellii,* harbouring a similar PaLoc to that of the classical Bi-toxin PaLoc of *C. difficile*^46^. Recently, Couchmam *et al.*^47^ found that a small number of *C. sordellii* strains carry PaLoc variants located on plasmids (pCS1-1 and pCS1-3, Fig. S7), substituting 14 genes of the pCS1 backbone. When we compared the gene content and organisation within the PaLoc of *C. sordellii* and *C. difficile* (Fig. S7), we observed in addition to the toxin similarity (*tcdB*/*tcsL* and *tcdA*/*tcsH*) that both species have the same PaLoc accessory genes, i.e., genes encoding a transcriptional regulator (*tcdR* and *tcsR*), a holin-like protein (*tcdE* and *tcsE*) and, when compared to the *C. difficile* strains CD10-165/SA10-050, an amidase (*cwlH*) (Fig. S7). Interestingly, among the described PaLoc variants, one has a truncated form of the *tcsH* gene, indicating possible genetic polymorphisms within the *C. sordellii* PaLoc region, as shown for *C. difficile*. Therefore, it seems reasonable to expand the *C. difficile* model to the PaLoc of *C. sordellii*, but more sequenced *C. sordellii* PaLoc types are required to prove it.

Together, this work supports a substantial body of evidence that argues for a scenario in which the classical “Bi-Toxin PaLoc” was generated by a fusion of two “Mono-Toxins PaLoc” (Fig. 6) and provides the basis for investigating the involved molecular mechanisms. In addition, the synteny between the *C. difficile* and *C. sordellii* PaLoc genes as reinforced by the presence of the *C. sordellii* holin-like gene *tcsE* in the PaLoc of the *C. difficile* strain ES130^15^ supports the idea of a common ancestor of the clostridial PaLoc.

## Additional Information

**How to cite this article**: Monot, M. *et al.* *Clostridium difficile*: New Insights into the Evolution of the Pathogenicity Locus. *Sci. Rep.* **5**, 15023; doi: 10.1038/srep15023 (2015).

## Supplementary Material

Supplementary Information

## Figures and Tables

**Table 1:** Analysis of RA09-70, SA10-050 and CD10-165 strains.

Toxin genes and toxin production	RA09-070	SA10-050	CD10-165
**A**			
PCR A1/A2/A3 (***tcdA)***	**+/+/−**	**−/−/−**	**−/−/−**
PCR B1/B2/B3 (***tcdB**)*	**−/−/−**	**+/+/−**	**+/+/−**
PCR binary toxin genes (***cdtA***, ***cdtB)***	**−**	**−**	**−**
PCR ***(tcdC***)	**−**	**−**	**−**
*illumigene C. difficile* (***tcdA***)	**−**	**−**	**−**
Rida Gene CD Toxin A/B (***tcdA***, ***tcdB***)	**+/+**	**+/−**	**+/−**
Xpert *C. difficile*, Cepheid (***tcdB***/***cdt***/***tcdC*** **del-117)**	**−/−/−**	**+/ −/−**	**+/−/−**
C. diff Quik Chek Complete, Alère **(GDH**/**TcdA, TcdB)**	+/+*	**+/+**	**+/+**
Vidas *C. difficile* **(TcdA, TcdB)**	**+**	**−**	**−**
CTA: CytoToxicity Assay **(TcdB)**	**−**	**+**	**+**
			
**Lemee** ***et al.***	**RA09-70**	**SA10-050**	**CD10-165**
**Genes**	**Allele (SNP)**	**Allele (SNP)**	**Allele (SNP)**
**B**			
aroE	19 (2)	14 (37)	14 (30)
dutA	13 (1)	**−**	**−**
gmk	9	9 (13)	9 (13)
groEL	17 (1)	10 (9)	10 (9)
recA	9	9 (9)	9 (9)
sodA	16 (1)	16 (29)	16 (27)
tpi	12	9 (9)	9 (9)
ST	**63**	**−**	**−**
			
**Griffiths** ***et al***.	**RA09-70**	**SA10-050**	**CD10-165**
**Genes**	**Allele (SNP)**	**Allele (SNP)**	**Allele (SNP)**
adk	15 (2)	13	13
atpA	21	18 (1)	18
dxr	28 (3)	22	22
glyA	38 (1)	40	31 (2)
recA	21 (1)	18 (2)	18
sodA	36	38	31
tpi	30	32 (2)	26
ST	**200**	**206**	**181**

(A) Detection of toxin gene and toxin production by different molecular methods and immuno assays. (B) MLST analysis according to the schemes of Lemee *et al.*^19^ and Griffiths *et al.*^20^.

^*^weak band; GDH: glutamate dehydrogenase.

**Table 2:** Amino acid identity of TcdR and CdtR among *C. difficile* strains.

Information			TcdR *amino acid identity* %			CdtR *amino acid identity* %	
Strain	Accession	Ribotype	CD630	RA09-70	SA10-050 CD10-165	CD630	SA10-050 CD10-165
CD630	AM180355	012	**100%**	75%	73%	**100%**	62%
RA09-70	PRJNA255280	–	75%	**100%**	70%	–	–
SA10-050 CD10-165	PRJNA260039 PRJNA260040	–	73%	70%	**100%**	62%	**100%**
Other Strains	*	001–577	>97%	74–75%	73–75%	>95%	59–62%

^*^Detailed in Supplementary Data Table S1.

## References

[b1] Rupnik M., Wilcox M. H. & Gerding D. N. Clostridium difficile infection: new developments in epidemiology and pathogenesis. Nature reviews. Microbiology 7, 526–536, 10.1038/nrmicro2164 (2009). 19528959

[b2] Wiegand P. N. *et al.* Clinical and economic burden of Clostridium difficile infection in Europe: a systematic review of healthcare-facility-acquired infection. The Journal of hospital infection 81, 1–14, 10.1016/j.jhin.2012.02.004 (2012). 22498638

[b3] Davies K. A. *et al.* Underdiagnosis of Clostridium difficile across Europe: the European, multicentre, prospective, biannual, point-prevalence study of Clostridium difficile infection in hospitalised patients with diarrhoea (EUCLID). The Lancet. Infectious diseases 14, 1208–1219, 10.1016/S1473-3099(14)70991-0 (2014). 25455988

[b4] Mani N. & Dupuy B. Regulation of toxin synthesis in Clostridium difficile by an alternative RNA polymerase sigma factor. Proc Natl Acad Sci USA 98, 5844–5849, 10.1073/pnas.101126598 (2001). 11320220 PMC33301

[b5] Govind R. & Dupuy B. Secretion of Clostridium difficile Toxins A and B Requires the Holin-like Protein TcdE. PLoS Pathog 8, e1002727, 10.1371/journal.ppat.1002727 (2012). 22685398 PMC3369941

[b6] Matamouros S., England P. & Dupuy B. Clostridium difficile toxin expression is inhibited by the novel regulator TcdC. Mol Microbiol 64, 1274–1288, 10.1111/j.1365-2958.2007.05739.x (2007). 17542920

[b7] Braun V., Hundsberger T., Leukel P., Sauerborn M. & von Eichel-Streiber C. Definition of the single integration site of the pathogenicity locus in Clostridium difficile. Gene 181, 29–38 (1996). 8973304 10.1016/s0378-1119(96)00398-8

[b8] Dingle K. E. *et al.* Evolutionary history of the Clostridium difficile pathogenicity locus. Genome biology and evolution 6, 36–52, 10.1093/gbe/evt204 (2014). 24336451 PMC3914685

[b9] Bauer M. P. *et al.* Clostridium difficile infection in Europe: a hospital-based survey. Lancet 377, 63–73, 10.1016/S0140-6736(10)61266-4 (2011). 21084111

[b10] Carter G. P. *et al.* Binary toxin production in Clostridium difficile is regulated by CdtR, a LytTR family response regulator. J Bacteriol 189, 7290–7301, 10.1128/JB.00731-07 (2007). 17693517 PMC2168464

[b11] Rupnik M., Avesani V., Janc M., von Eichel-Streiber C. & Delmee M. A novel toxinotyping scheme and correlation of toxinotypes with serogroups of Clostridium difficile isolates. J Clin Microbiol 36, 2240–2247 (1998). 9665999 10.1128/jcm.36.8.2240-2247.1998 PMC105025

[b12] Rupnik M. *et al.* Revised nomenclature of Clostridium difficile toxins and associated genes. J Med Microbiol 54, 113–117 (2005). 15673503 10.1099/jmm.0.45810-0

[b13] Popoff M. R. & Bouvet P. Genetic characteristics of toxigenic Clostridia and toxin gene evolution. Toxicon: official journal of the International Society on Toxinology 75, 63–89, 10.1016/j.toxicon.2013.05.003 (2013). 23707611

[b14] Stabler R. A. *et al.* Comparative phylogenomics of Clostridium difficile reveals clade specificity and microevolution of hypervirulent strains. J Bacteriol 188, 7297–7305, 10.1128/JB.00664-06 (2006). 17015669 PMC1636221

[b15] Elliott B., Dingle K. E., Didelot X., Crook D. & Riley T. V. The complexity and diversity of the Pathogenicity locus in Clostridium difficile clade 5. Genome biology and evolution, 10.1093/gbe/evu248 (2014). PMC4986448 25381663

[b16] Janezic S., Marin M., Martin A. & Rupnik M. A new type of toxin A-negative, toxin B-positive Clostridium difficile strain lacking a complete tcdA gene. J Clin Microbiol, 10.1128/JCM.02211-14 (2014). PMC4298534 25428159

[b17] Eckert C. *et al.* Clinical and microbiological features of Clostridium difficile infections in France: the ICD-RAISIN 2009 national survey. Medecine et maladies infectieuses 43, 67–74, 10.1016/j.medmal.2013.01.004 (2013). 23498135

[b18] Bidet P., Barbut F., Lalande V., Burghoffer B. & Petit J. C. Development of a new PCR-ribotyping method for Clostridium difficile based on ribosomal RNA gene sequencing. FEMS microbiology letters 175, 261–266 (1999). 10386377 10.1111/j.1574-6968.1999.tb13629.x

[b19] Lemee L. *et al.* Multilocus sequence analysis and comparative evolution of virulence-associated genes and housekeeping genes of Clostridium difficile. Microbiology 151, 3171–3180, 10.1099/mic.0.28155-0 (2005). 16207902

[b20] Griffiths D. *et al.* Multilocus sequence typing of Clostridium difficile. J Clin Microbiol 48, 770–778, 10.1128/JCM.01796-09 (2010). 20042623 PMC2832416

[b21] Lalande V. *et al.* Evaluation of a loop-mediated isothermal amplification assay for diagnosis of Clostridium difficile infections. J Clin Microbiol 49, 2714–2716, 10.1128/JCM.01835-10 (2011). 21525213 PMC3147856

[b22] Kurka H. *et al.* Sequence similarity of Clostridium difficile strains by analysis of conserved genes and genome content is reflected by their ribotype affiliation. PLoS One 9, e86535, 10.1371/journal.pone.0086535 (2014). 24482682 PMC3902958

[b23] Zerbino D. R. & Birney E. Velvet: algorithms for *de novo* short read assembly using de Bruijn graphs. Genome Res 18, 821–829, 10.1101/gr.074492.107 (2008). 18349386 PMC2336801

[b24] Vallenet D. *et al.* MicroScope: a platform for microbial genome annotation and comparative genomics. Database (Oxford) 2009, bap021, 10.1093/database/bap021 (2009). PMC2790312 20157493

[b25] Tamura K., Stecher G., Peterson D., Filipski A. & Kumar S. MEGA6: Molecular Evolutionary Genetics Analysis version 6.0. Molecular biology and evolution 30, 2725–2729, 10.1093/molbev/mst197 (2013). 24132122 PMC3840312

[b26] Dineen S. S., Villapakkam A. C., Nordman J. T. & Sonenshein A. L. Repression of Clostridium difficile toxin gene expression by CodY. Mol Microbiol 66, 206–219, 10.1111/j.1365-2958.2007.05906.x (2007). 17725558

[b27] Antunes A. *et al.* Global transcriptional control by glucose and carbon regulator CcpA in Clostridium difficile. Nucleic Acids Res, 10.1093/nar/gks864 (2012). PMC3510511 22989714

[b28] Dupuy B. & Matamouros S. Regulation of toxin and bacteriocin synthesis in Clostridium species by a new subgroup of RNA polymerase sigma-factors. Res Microbiol 157, 201–205, 10.1016/j.resmic.2005.11.004 (2006). 16439101

[b29] El Meouche I. *et al.* Characterization of the SigD regulon of C. difficile and its positive control of toxin production through the regulation of tcdR. PLoS One 8, e83748, 10.1371/journal.pone.0083748 (2013). 24358307 PMC3865298

[b30] Sao-Jose C. *et al.* Diversity in the lysis-integration region of oenophage genomes and evidence for multiple tRNA loci, as targets for prophage integration in Oenococcus oeni. Virology 325, 82–95, 10.1016/j.virol.2004.04.029 (2004). 15231388

[b31] Smith D. L. & Young R. Oligohistidine tag mutagenesis of the lambda holin gene. J Bacteriol 180, 4199–4211 (1998). 9696770 10.1128/jb.180.16.4199-4211.1998 PMC107418

[b32] Squire M. M. *et al.* Novel molecular type of Clostridium difficile in neonatal pigs, Western Australia. Emerg Infect Dis 19, 790–792, 10.3201/eid1905.121062 (2013). 23697508 PMC3647499

[b33] Elliott B. *et al.* New types of toxin A-negative, toxin B-positive strains among clinical isolates of Clostridium difficile in Australia. J Med Microbiol 60, 1108–1111, 10.1099/jmm.0.031062-0 (2011). 21393460

[b34] Garnier T. & Cole S. T. Complete nucleotide sequence and genetic organization of the bacteriocinogenic plasmid, pIP404, from Clostridium perfringens. Plasmid 19, 134–150 (1988). 2901768 10.1016/0147-619x(88)90052-2

[b35] Regamey A. & Karamata D. The N-acetylmuramoyl-L-alanine amidase encoded by the Bacillus subtilis 168 prophage SP beta. Microbiology 144(Pt 4) 885–893 (1998). 9579063 10.1099/00221287-144-4-885

[b36] Wang I. N., Smith D. L. & Young R. Holins: the protein clocks of bacteriophage infections. Annual review of microbiology 54, 799–825, 10.1146/annurev.micro.54.1.799 (2000). 11018145

[b37] Didelot X. *et al.* Microevolutionary analysis of Clostridium difficile genomes to investigate transmission. Genome Biol 13, R118, 10.1186/gb-2012-13-12-r118 (2012). 23259504 PMC4056369

[b38] Eyre D. W. *et al.* Diverse sources of C. difficile infection identified on whole-genome sequencing. N Engl J Med 369, 1195–1205, 10.1056/NEJMoa1216064 (2013). 24066741 PMC3868928

[b39] Bouvet P. J. & Popoff M. R. Genetic relatedness of Clostridium difficile isolates from various origins determined by triple-locus sequence analysis based on toxin regulatory genes tcdC, tcdR, and cdtR. J Clin Microbiol 46, 3703–3713, 10.1128/JCM.00866-08 (2008). 18832125 PMC2576625

[b40] Carter G. P. *et al.* TcsL is an essential virulence factor in Clostridium sordellii ATCC 9714. Infect Immun 79, 1025–1032, 10.1128/IAI.00968-10 (2011). 21199912 PMC3067498

[b41] Rood J. I. & Cole S. T. Molecular genetics and pathogenesis of Clostridium perfringens. Microbiol Rev 55, 621–648 (1991). 1779929 10.1128/mr.55.4.621-648.1991 PMC372840

[b42] Anthony T., Chellappa G. S., Rajesh T. & Gunasekaran P. Functional analysis of a putative holin-like peptide-coding gene in the genome of Bacillus licheniformis AnBa9. Archives of microbiology 192, 51–56, 10.1007/s00203-009-0530-7 (2010). 19967339

[b43] Dingle K. E. *et al.* Clinical Clostridium difficile: clonality and pathogenicity locus diversity. PLoS One 6, e19993, 10.1371/journal.pone.0019993 (2011). 21625511 PMC3098275

[b44] Monot M. *et al.* Reannotation of the genome sequence of Clostridium difficile strain 630. J Med Microbiol 60, 1193–1199, 10.1099/jmm.0.030452-0 (2011). 21349987

[b45] Brouwer M. S. *et al.* Horizontal gene transfer converts non-toxigenic Clostridium difficile strains into toxin producers. Nature communications 4, 2601, 10.1038/ncomms3601 (2013). PMC3826655 24131955

[b46] Sirigi Reddy A. R., Girinathan B. P., Zapotocny R. & Govind R. Identification and characterization of Clostridium sordellii toxin gene regulator. J Bacteriol 195, 4246–4254, 10.1128/JB.00711-13 (2013). 23873908 PMC3754755

[b47] Couchman E. C. *et al.* Clostridium sordellii genome analysis reveals plasmid localized toxin genes encoded within pathogenicity loci. BMC genomics 16, 392, 10.1186/s12864-015-1613-2 (2015). 25981746 PMC4434542

[b48] Brudno M. *et al.* LAGAN and Multi-LAGAN: efficient tools for large-scale multiple alignment of genomic DNA. Genome Res 13, 721–731, 10.1101/gr.926603 (2003). 12654723 PMC430158

[b49] Mayor C. *et al.* VISTA : visualizing global DNA sequence alignments of arbitrary length. Bioinformatics 16, 1046–1047 (2000). 11159318 10.1093/bioinformatics/16.11.1046

[b50] Altschul S. F., Gish W., Miller W., Myers E. W. & Lipman D. J. Basic local alignment search tool. J Mol Biol 215, 403–410, 10.1016/S0022-2836(05)80360-2 (1990). 2231712

[b51] Monot M., Orgeur M., Camiade E., Brehier C. & Dupuy B. COV2HTML: a visualization and analysis tool of bacterial next generation sequencing (NGS) data for postgenomics life scientists. Omics: a journal of integrative biology 18, 184–195, 10.1089/omi.2013.0119 (2014). 24512253 PMC3934542

