# Supplementary material for: Clostridium difficile: New Insights into the Evolution of the Pathogenicity Locus
Source: Sci Rep. 2015 Oct 8;5:15023. doi: 10.1038/srep15023 (PMC4597214; doi:10.1038/srep15023)

Clostridium difficile: New Insights into the Evolution of the Pathogenicity Locus (Supplementary Data)

Marc Monot[1*], Catherine Eckert[3,4], Astrid Lemire[3,4], Audrey Hamiot[1], Thomas Dubois[1], Carine Tessier[3,4], Bruno Dumoulard[5], Benjamin Hamel[6], Amandine Petit[3,4], Valérie Lalande[3,4], Laurence Ma[2], Christiane Bouchier[2], Frédéric Barbut[3,4,A] and Bruno Dupuy[1,A]

Case reports

RA09-070 strain was isolated from a patient with an antibiotic-associated diarrhea. This 60-old-year man was admitted to Cambrai Hospital, Cambrai, France on 11 February 2009 for hepatic decompensation with jaundice and confusion suggesting cholecystitis and acute and severe alcoholic hepatitis. He was febrile (38.2°C), the white blood cell count was 4,300/mm^3; the C-reactive protein value was 108 mg/L. An antimicrobial treatment for inflammatory syndrome and suspicion of fever from digestive origin, associating amoxicillin and clavulanic acid was initiated and then replaced by cefotaxim (1 g three times a day, IV), ofloxacin (200 mg twice a day, per os) and metronidazole (500 mg three times a day, per os) from the 13 to 25 February. Amikacin (1 g per day, IV) was added from the 13 to 18 February. The antibiotic treatment was modified for ciprofloxacin (400 mg twice a day, IV) on 26 February. Piperacillin-tazobactam (4 g four times a day, IV, 28 February to 05 March) and gentamycin (80 mg twice a day, IV, 26 to 27 February) were also added. On 28 February, the patient was transferred to an intensive care unit for respiratory decompensation. Proton pump inibitors (pantoprazole, 20 mg per day and esomeprazole, 40 mg twice a day) were also given successively from the 12 February until the patient was discharged on 29 April. The patient with improved clinical outcome was transferred again to gastroenterology ward on 5 March and antibiotics were stopped on 16 March. On 21 March, the expectoration was positive to *Candida albicans* and due to loss of body weight and condition, fluconazole was given to the patient. A colitis due to *C. difficile* was diagnosed 8 days later, on 29 March using the Meridian tox A/B assay and the specific culture medium CLO (Biomérieux). The patient was treated by metronidazole 250 mg four times a day for 2 days and then by metronidazole 500 mg three times a day, per os for 13 days. Diarrhea resolved on 1st April. The patient did not present recurrence.

SA10-050 strain was isolated from a 84 old-year man admitted in Saint Antoine hospital, Paris, France, on 8 April 2010 for relapse of multiple myeloma. Treatments by pomalidomide

(4 mg per day) and dexamethasone (40 mg per week) were started on 27 April. Esomeprazole (80 mg IV per day) was also started. On 13 May pomalidomide is stopped for cytopenia. On 17 May pomalidomide is re-introduced at 2 mg per day until 1st June and was stopped again for cytopenia. On 10 May amoxicillin-clavulanic acid was given for inflammatory syndrome and replaced by tazocillin, ciprofloxacin and trimethoprim-sulfamethoxazole on 27 May leading to an improvement of the symptoms. Domperidone and loperamide were started on 23 May and stopped on 16 June. On 27 May, the patient presented with febrile diarrhea (5-7 bowel movements per day). The C-reactive protein value was 276 mg/L. *C. difficile* infection was diagnosed on 3 June using stool cytotoxicity assay and toxigenic culture and was treated by metronidazole (500 mg twice a day for 10 days (7 June to 16 June) given by nasogastric tube. On 2 June, worsening respiratory symptoms were observed and pneumocystose was diagnosed: trimethoprim/sulfamethoxazole and voriconazole were started. On 17 June the patient is discharged to palliative care.

CD10-165 strain was isolated from a patient admitted in Villefranche-sur-Saône hospital, Villefranche-sur-Saône, France on 13 July 2010. This 74 old-year man was previously hospitalized from 19 March to 8 April for acute decompensated heart failure due to erysipelas treated by amoxicillin 1 g three times a day for 14 days. This patient was also treated by rabeprazole (10 mg per day). After discharge, the patient was hospitalized again on 20 May in pneumology for decompensation of chronic obstructive pulmonary disease. The patient was treated with prednisolone (60 mg to 20 mg). After discharge, the patient presented with diarrhea and asthenia which led to a third hospitalization of the patient on 13 July. Patient was treated with amoxicillin and clavulanic acid (1 g three times a day) for an infectious syndrome and decompensation of chronic obstructive pulmonary disease. On admission the patient was febrile (37.8°C), the C-reactive protein value was 184 mg/L, white blood cell count was 25 000/mm^3, the lactate value was 0.9 mmol/L and the creatinine value was 118 µmol/L. The day after the patient worsened, his abdomen was distended and diarrhea was abundant. The C-reactive protein, the white blood cell count, the lactate and creatinine values increased to 298 mg/L, 51 000/mm^3, 10 mmol/L and 175 µmol/L, respectively. The scanner performed showed a peritoneal effusion with a thickening aspect of the sigmoid and the colon. A colostomy was performed and pseudomembrans were found. The patient deceased on 14 July in a context of a multiple organ failure and shock. The immune-enzymatic test (Immunocard, Meridian) performed on 15 July was positive for the toxins A/B in a stool sample. The death was directly attributed to *C. difficile*.

Table S1. TcdR and CdtR *C. difficile* homology between strains

Information					TcdR amino acid identity %			CdtR amino acid identity %	
**Strain**	**Accession**	**Ribotype**	**MLST** [1]	**Clade**	**CD630**	**RA09-70**	**SA10-050 CD10-165**	**CD630**	**SA10-050 CD10-165**
CD630	AM180355	012	54	1	**100%**	75%	73%	**100%**	62%
RA09-70	PRJNA255280	-	~200	5	75%	**100%**	70%	-	-
SA10-050<br>CD10-165	PRJNA260039<br>PRJNA260040	-	~206<br>~181	C-I	73%	70%	**100%**	62%	**100%**
E23	PRJEB211	001	3	1	100%	75%	73%	100%	62%
E25	PRJEB196	005	6	1	100%	75%	73%	100%	62%
E16	PRJEB194	015	7	1	100%	75%	73%	-	-
R20291	FN545816.1	027	1	2	100%	75%	73%	96%	59%
E15	PRJEB199	075	95	2	100%	75%	73%	96%	59%
T6	PRJEB204	095	13	1	100%	75%	73%	100%	62%
E12	PRJEB212	106	42	1	100%	75%	73%	100%	62%
T14	PRJEB215	156	42	1	100%	75%	73%	100%	62%
T10	PRJEB214	165	3	1	100%	75%	73%	100%	62%
E14	PRJEB205	014	2	1	99%	74%	73%	100%	62%
CF5	FN665652.1	017	86	4	97%	74%	73%	-	-
T23	PRJEB200	019	67	2	99%	75%	74%	96%	59%
E24	PRJEB202	020	2	1	99%	74%	73%	100%	62%
T17	PRJEB206	025	49	1	99%	74%	73%	100%	62%
T20	PRJEB189	078	11	5	99%	75%	73%	-	-
T5	PRJEB188	079	11	5	99%	75%	73%	-	-
E1	PRJEB190	126	11	5	99%	75%	73%	-	-
E19	PRJEB201	577	62	2	98%	75%	75%	96%	59%
Ox1232*	PRJEB3093	-	25	3	97%	74%	73%	95%	60%
Ox2183*	PRJEB1483	-	5	3	97%	74%	73%	95%	61%
OxB_HB21*	PRJEB1483	-	30	4	-	-	-	-	-
Q24*	PRJEB1483	-	162	3	97%	74%	74%	95%	61%
ES248*	PRJEB1483	-	170	4	97%	74%	73%	-	-
H5078*	PRJEB1483	-	177	C-I	-	-	-	-	-
RPH118*	PRJEB1483	-	178	C-I	-	-	-	-	-
RPH97*	PRJEB1483	-	179	C-I	-	-	-	-	-
Ox561*	PRJEB1729	-	22	3	97%	74%	73%	95%	61%
Oxl_WBC201l*	PRJEB1483	-	39	4	-	-	-	-	-

** Rawdata reads only, genome unavalaible.*

1 Griffiths, D. *et al*. Multilocus sequence typing of Clostridium difficile. *J Clin Microbiol* **48**, 770-778 (2010).

2 Bidet, P., Barbut, F., Lalande, V., Burghoffer, B. & Petit, J. C. Development of a new PCR-ribotyping method for Clostridium difficile based on ribosomal RNA gene sequencing. *FEMS microbiology letters* **175**, 261-266 (1999).

Figure S1. Caracteristic of the three strains

TcdA Dot Blot analysis of CD10-165, SA10-050 and RA09-70 strains

630Δ*erm* *tcdA*⁻	630Δ*erm*	CD10-165	SA10-050	RA09-070

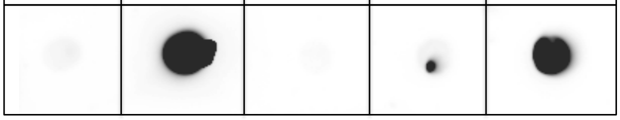

TcdA Dot blot analysis: Crude lysates (100ng) of strains were probed with TcdA monoclonal antibody (PCG4). 630Δ*erm* and its *tcdA*⁻ mutant (a generous gift of Sarah Kuehne, University Nottingham) and were used as positive and negative controls, respectively

PCR-ribotypage according to Bidet *et al.*[2]

Agarose Gel

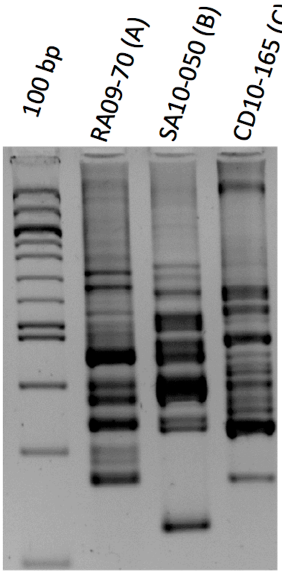

Capillary

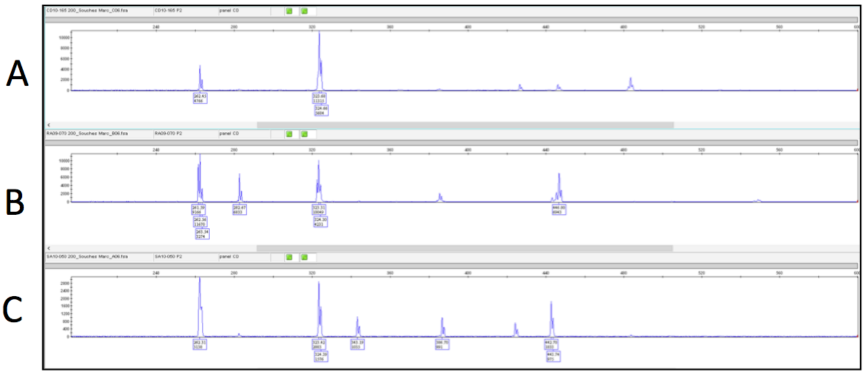

Figure S2. PCR validation of the PaLoc genomic location of the RA09-70 strain and of the organisation of the PaLoc-CdtLoc of SA10-050 and CD10-165 strains

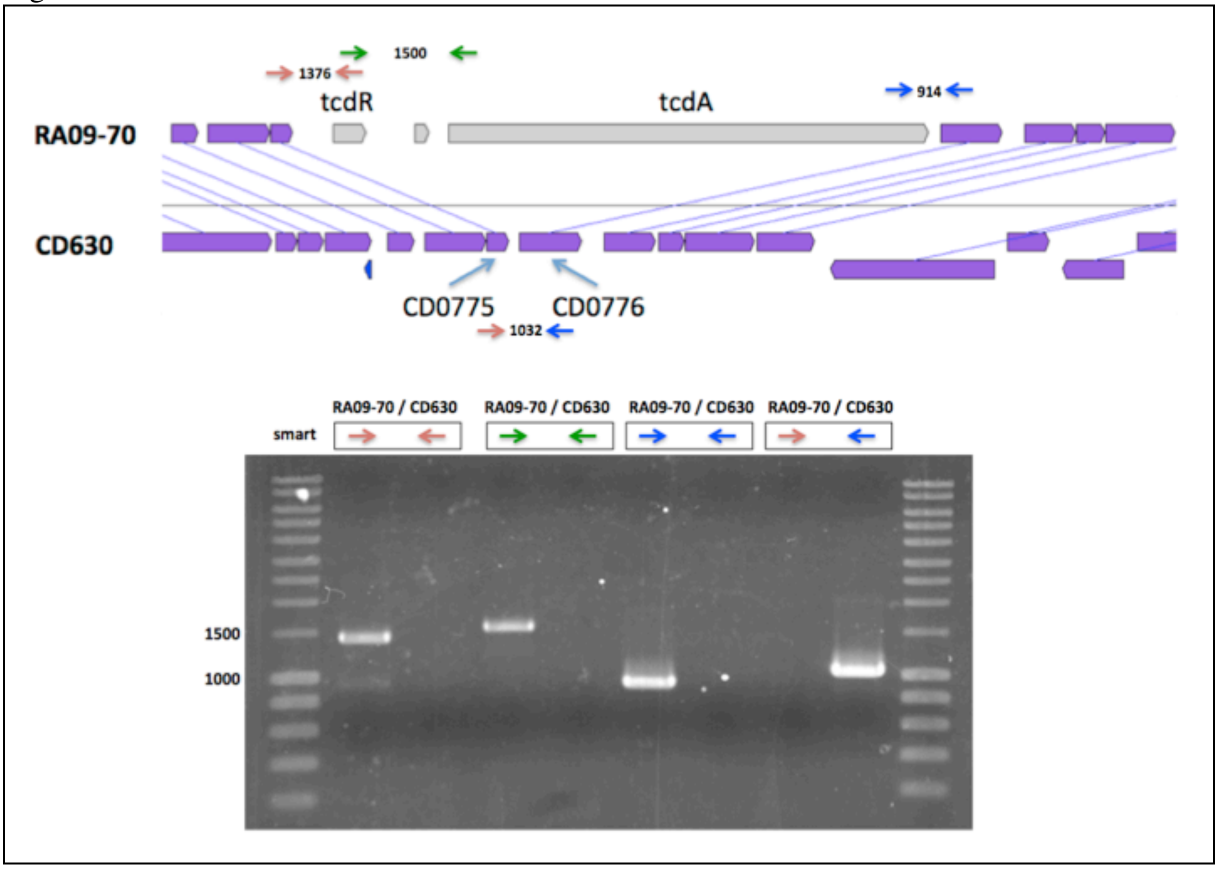

SA10-050 and CD10-165

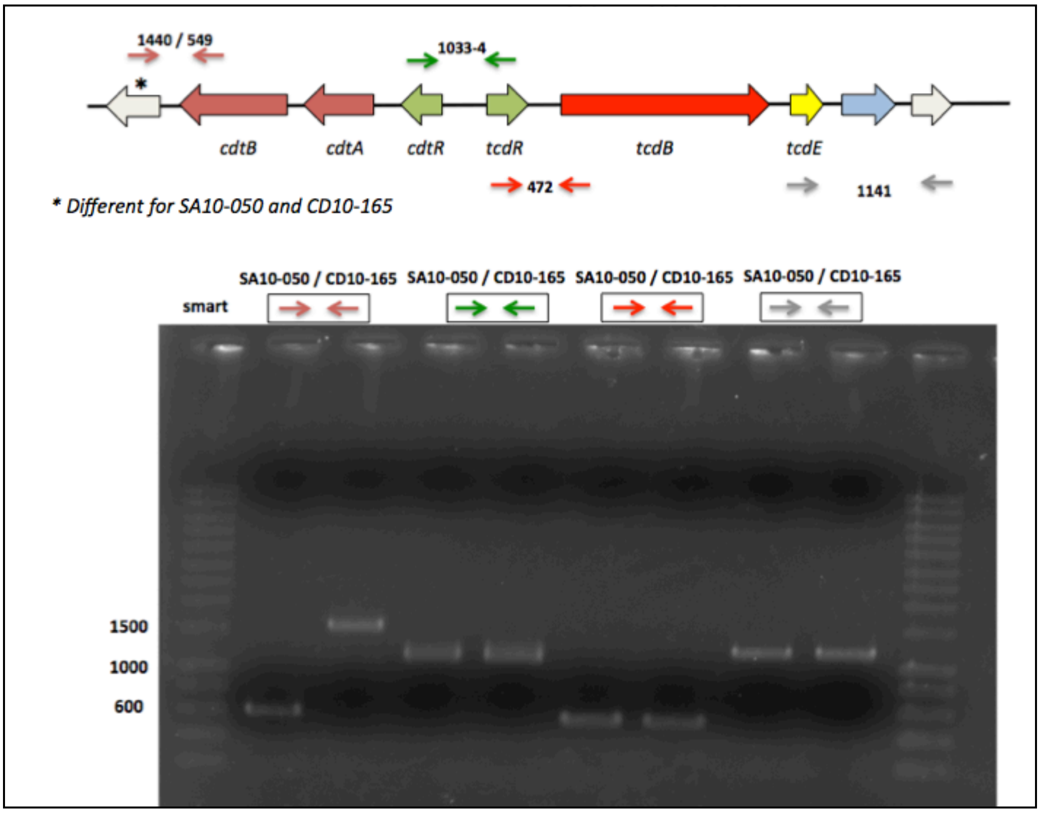

Figure S3. Maximum likehood tree constructed from *cdd3* gene

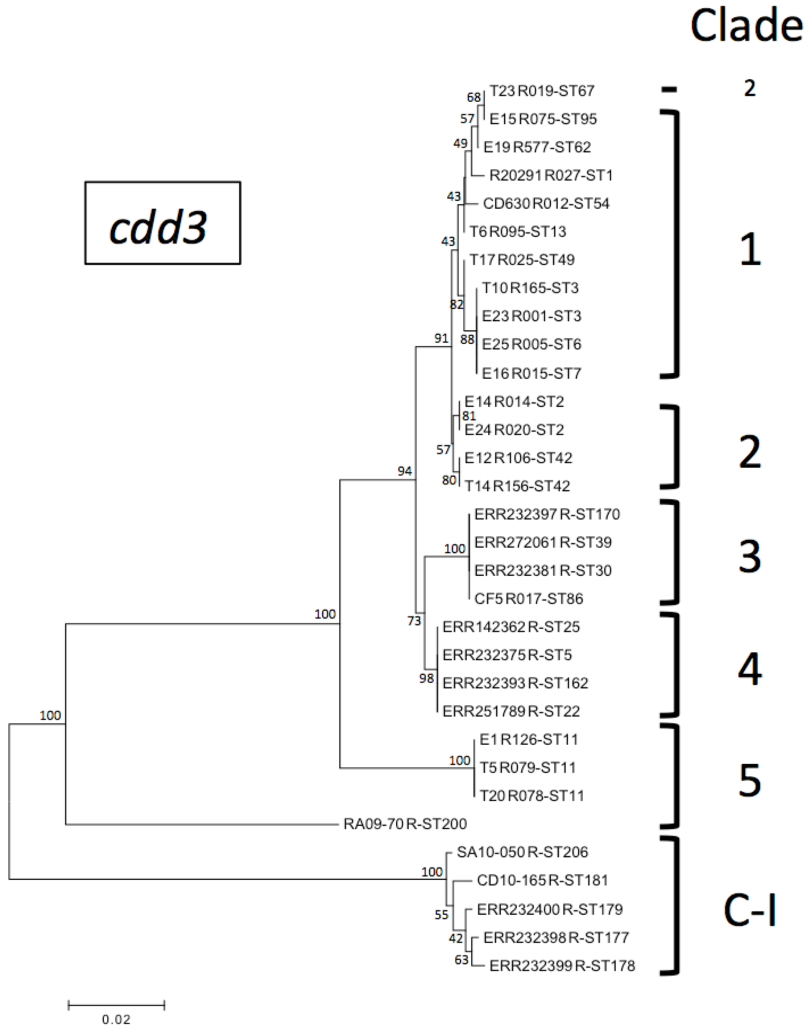

Figure S4. Analysis of UviB

TMHMM prediction of transmembrane segments for UviB

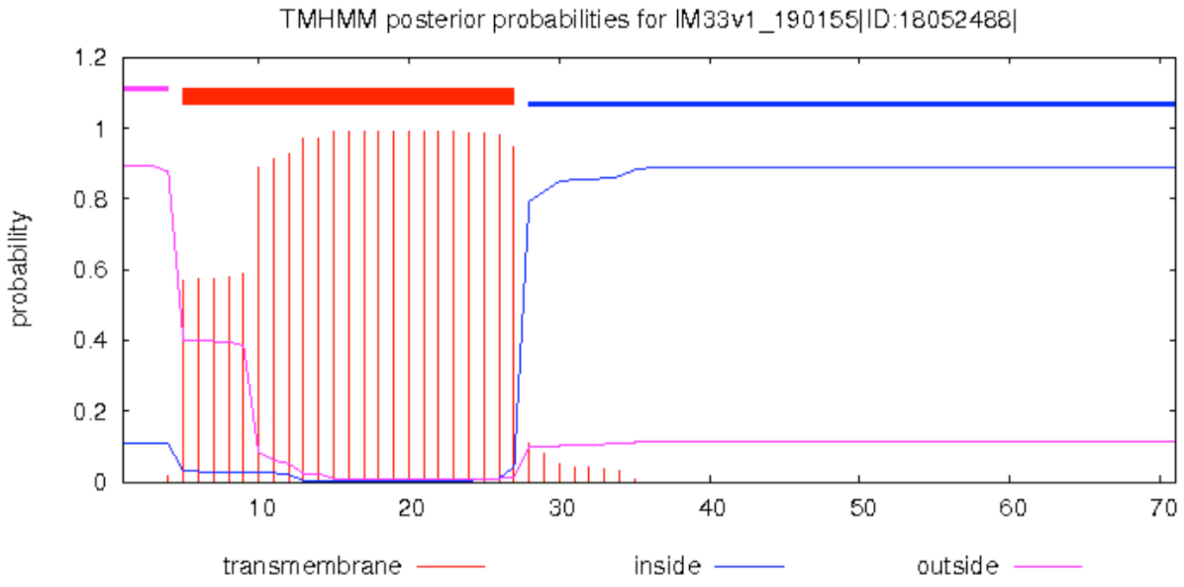

Dendogram of UviB/BhlA proteins from Clostridia and Bacillus species

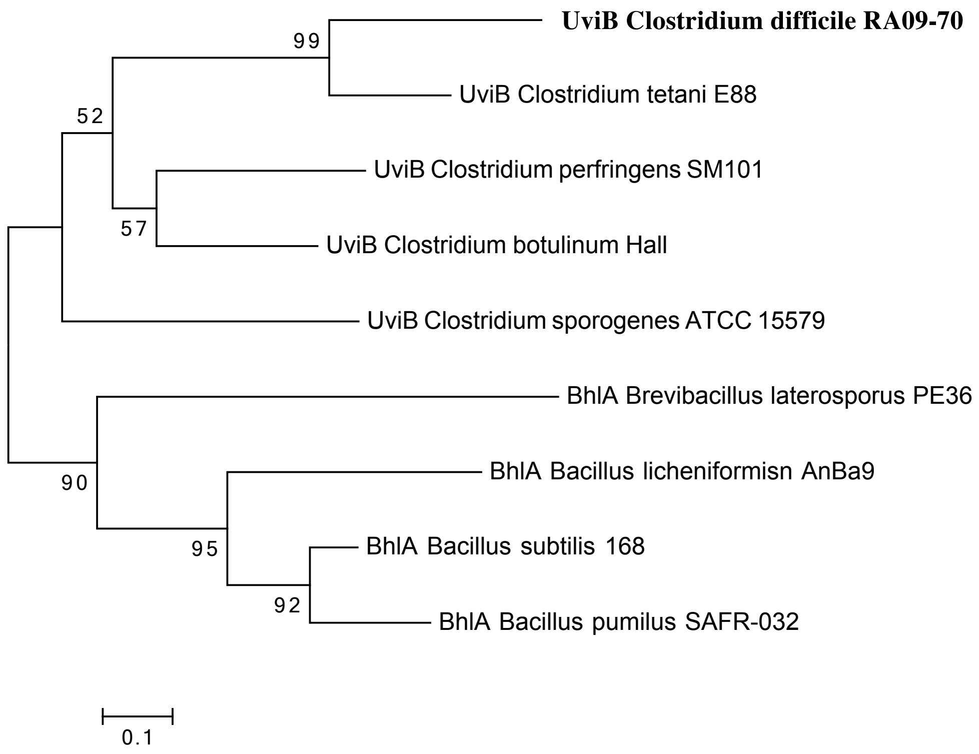

Figure S5. Sequence Alignments between autolysin and UviB genes and the *tcdE-tcdA* intergenic region of the CD630 PaLoc

With autolysin gene downstream of the *tcdE* gene of SA10-050 / CD10-165 strains

```
- 1 - [Clostridium difficile 630 chromosome NC_009089]
- Left-hand Genomic object:   CD630_06610 | +2 | 794615-795115 | tcdE | Holin-like pore-forming protein
- Overlapping Genomic object: CD630_06620 | +2 | 795224-795355 | Fragment of phage endolysin
- Right-hand Genomic object:  CD630_06630 | +3 | 795843-803975 | tcdA | Toxin A
LD= 4290252 LQ= 759 L= 317 Id= 80.00 %

Q:          1  ATGGTAGATATAATAAAGATGTTAACAAATAAAAAGTGCTATCCAAACAAAAACAATGCA  60
               |||||||||||||||||||| ||||||||||||||||| |||||||| || || |    |
S:     795119  ATGGTAGATATAATAAAGATATTAACAAATAAAAAGTGTTATCCAAATAAGAATAGCTGA  795178

Q:         61  AAGTTTATAGTAATTCATGAAACTGACAACGAGAGCAAGGGAGCAAATGCTAAAAGACAT  120
               ||||| ||  |||||||||||||| | || || | | |||||||| |||| || |||||
S:     795179  AAGTT-ATCATAATTCATGAAACTAATAATGAAAACGAGGGAGCAGATGCCAAGAGACA-  795236

Q:        121  GCACAAGCATTAAATGCAGGCAACTTAGAAGCAAGTGTCCATTACTATGTAGATGATAAG  180
                |||||| ||||||| ||   || || ||||||||||| ||||||||| ||||||| |||
S:     795237  -CACAAGTATTAAATACATATAATTTCGAAGCAAGTGTTCATTACTATATAGATGACAAG  795295

Q:        181  GTGATATATCAAACATTAGACCACAAAAACGGAGCATGGGCAGTTGGCAAAAGCTATTAA  237
               ||  ||||||||||||| |  |||||| | || |||||| ||||||| |||| ||||
S:     795296  GTAGTATATCAAACATTGGTTCACAAAGATGGTGCATGGTCAGTTGGTAAAATCTATGGG  795355

Q:        238  GCTACATTAGTTACAGATATCACAAACTATAATAGT-TAAACATAGAAATATGTGTAAAT  296
                || || || |  |||  || |||||||| |||||| |||||||||||||||||||||||
S:     795356  ACTGCACTAATCGCAGGAGTAACAAACTACAATAGTATAAACATAGAAATATGTGTAAAT  795415

Q:        297  AAAGATGGAAATTATACAAAA  317
                  |||||||||||| |||||
S:     795416  TGTGATGGAAATTATTCAAAA  795436
```

With the *uviB* gene upstream of the *tcdA* gene of the RA09-70 strain

```
- 1 - [Clostridium difficile 630 chromosome NC_009089]
- Left-hand Genomic object:   CD630_06620 | +2 | 795224-795355 | Fragment of phage endolysin
- Right-hand Genomic object:  CD630_06630 | +3 | 795843-803975 | tcdA | Toxin A
LD= 4290252 LQ= 216 L= 136 Id= 85.29 %

Q:         81  AAAAACACAAAAAATATTTGCTGAAAGGCAAAATAATAGGGAAAATAATTATCAAATTTT  140
               ||||||||||||| ||  || |||| | |||||| |||  |||||||| |||||||  ||
S:     795432  AAAAACACAAAAA-TACGTGATGAAGGACAAAATGATATAGAAAATAAGTATCAAACCTT  795490

Q:        141  AATAAGGGATTTAACTGATAGATTAAAAGTTACAGAAAAAATATATAAAGAATAAAAAA  200
               |||||  ||||||| |||||| |||||||||| || ||||||||||||||||||||||||
S:     795491  AATAAATGATTTAATTGATAGTTTAAAAGTTATAGGAAAAATATATAAAGAATAAAAAC  795550

Q:        201  TATTAAAAAAATATAA  216
                |||||||||||||||
S:     795551  -ATTAAAAAAATATAA  795565
```

Figure S6. Genetic organisation of tcdR promoter region

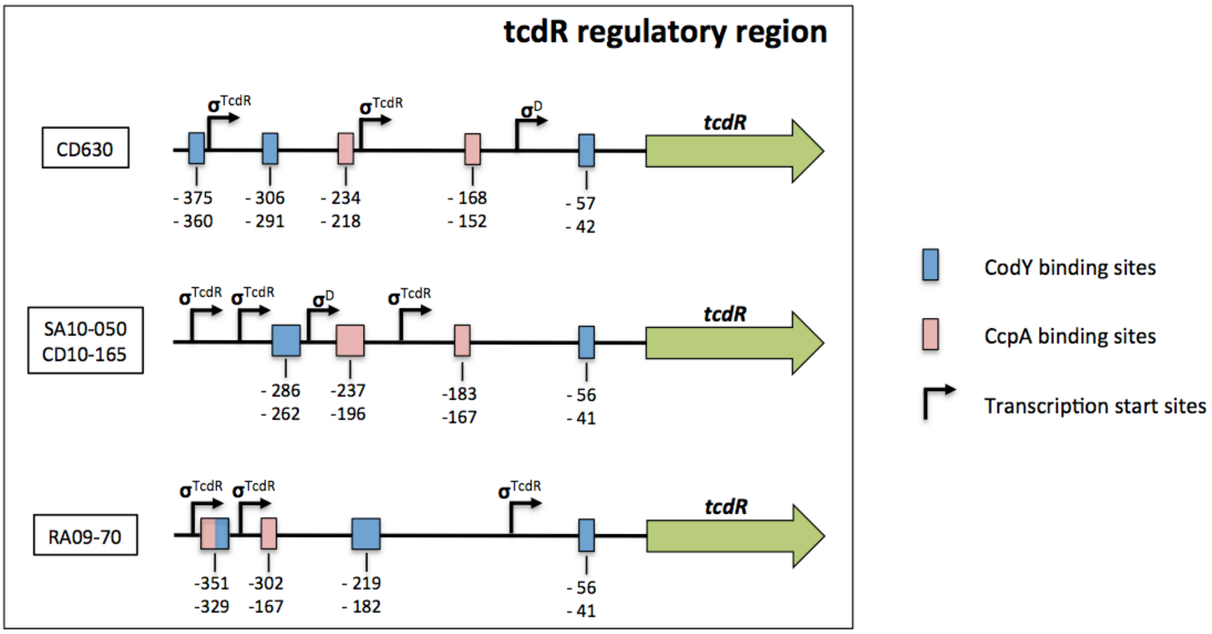

Position indicated are relative to the ATG start codon.

Figure S7. Types of *C. difficile* and *C. sordellii* pathogenicity locus

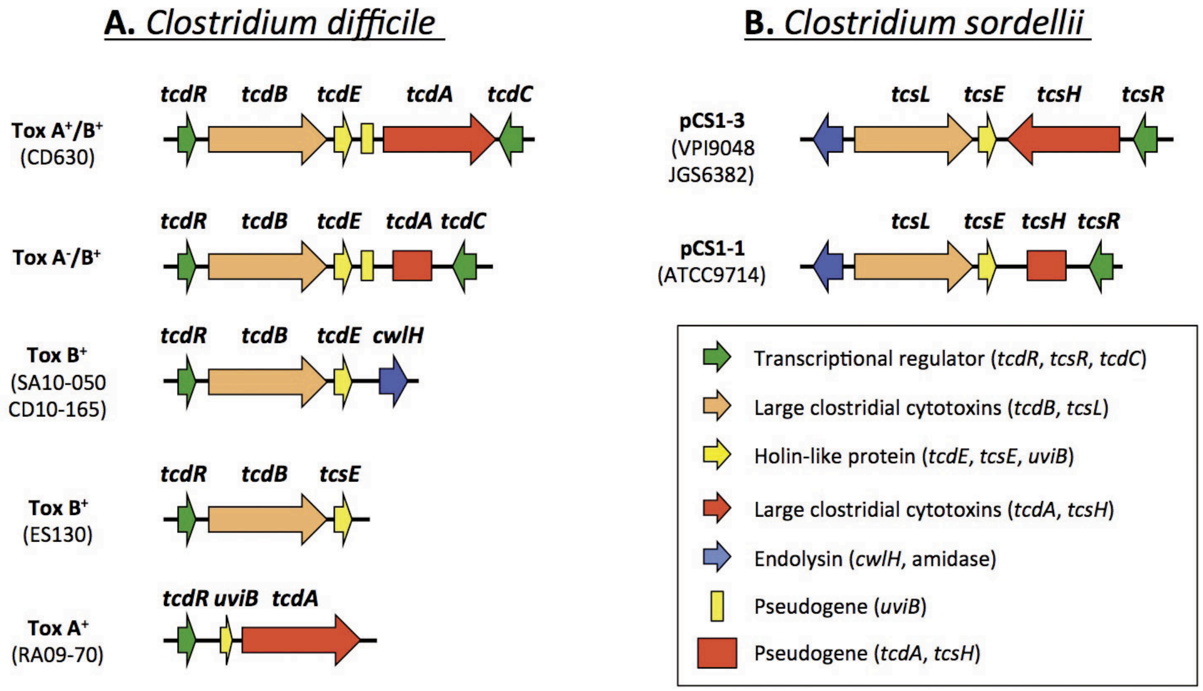

Figure S8. Analysis of TcdA (cf. Material and Methods)

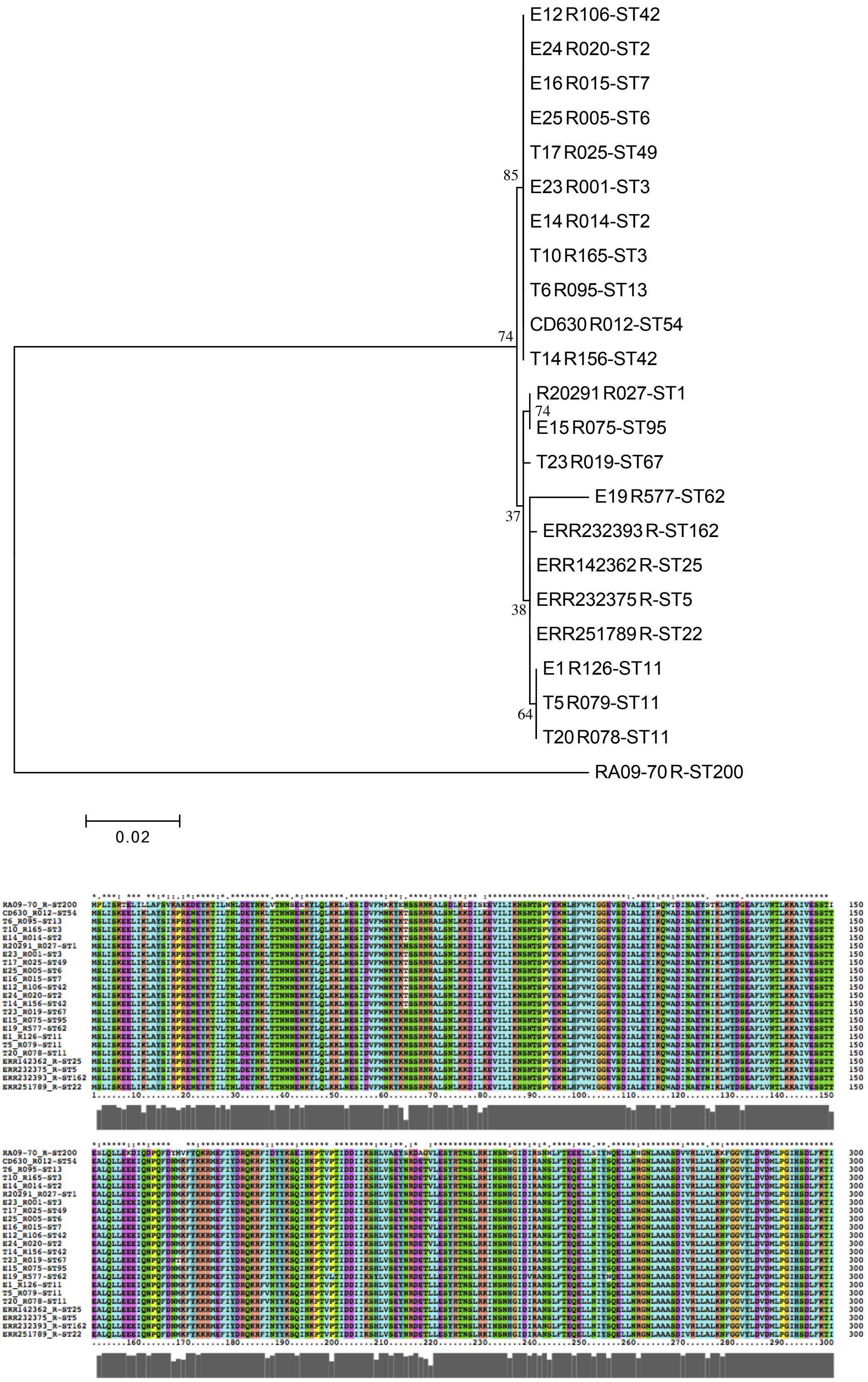

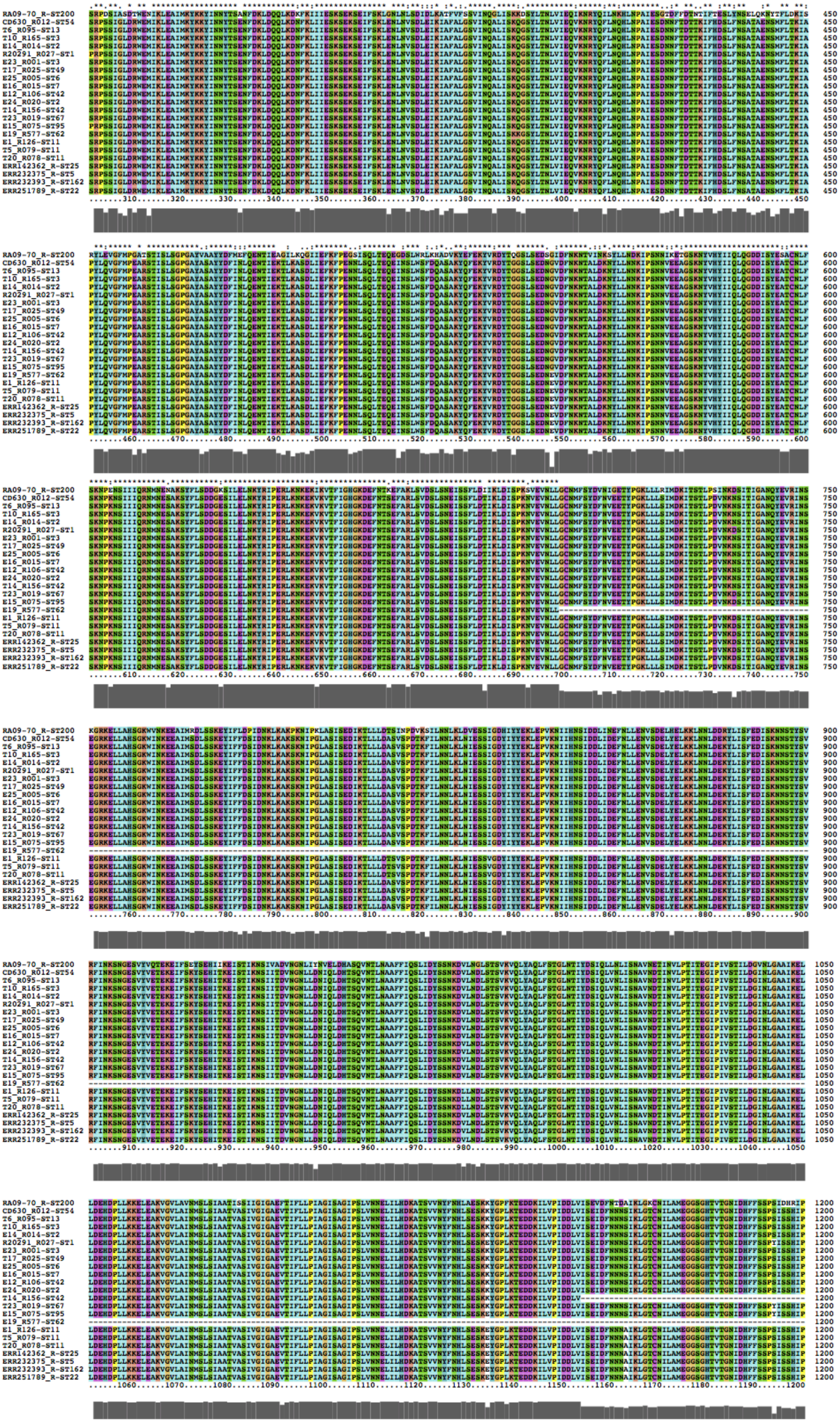

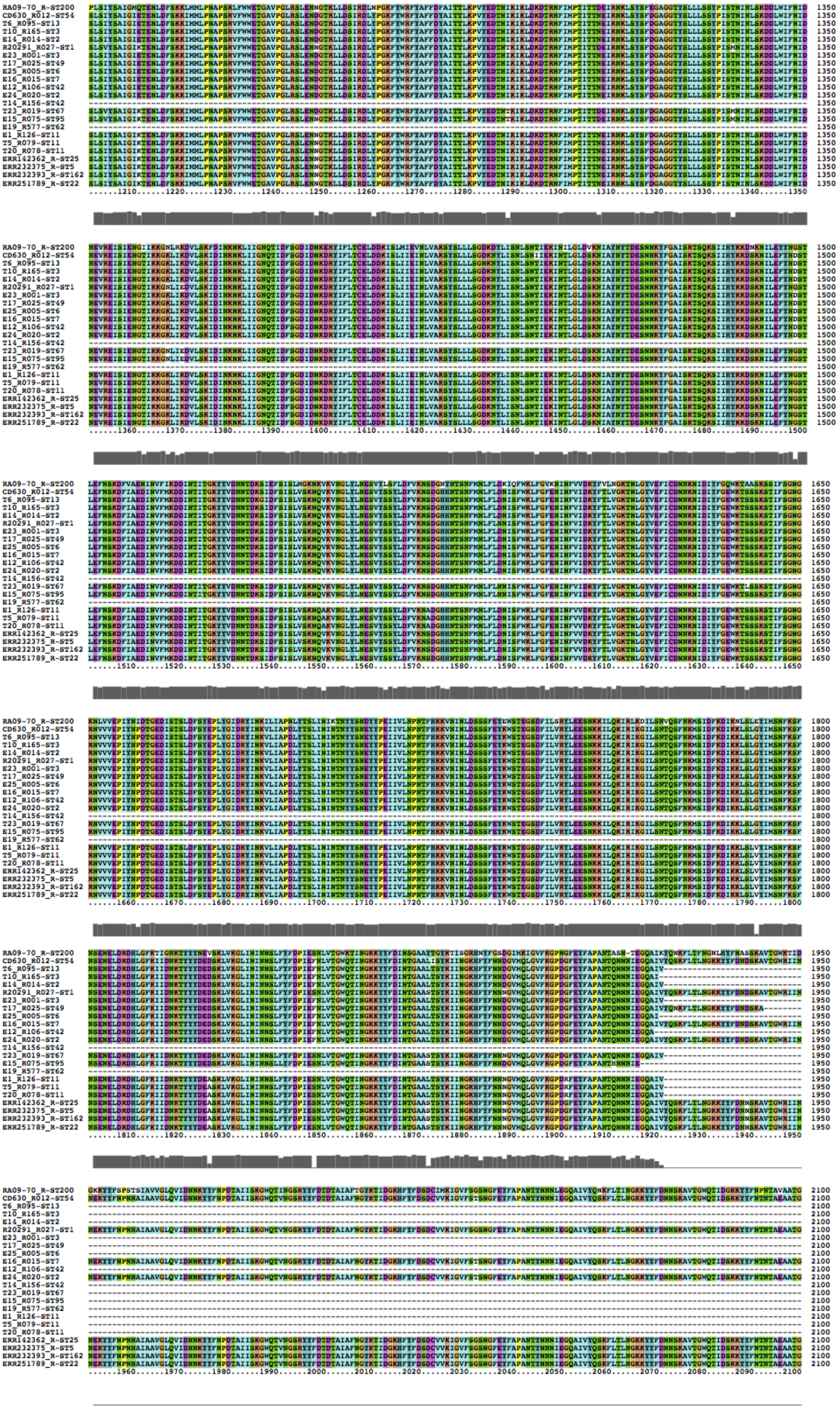

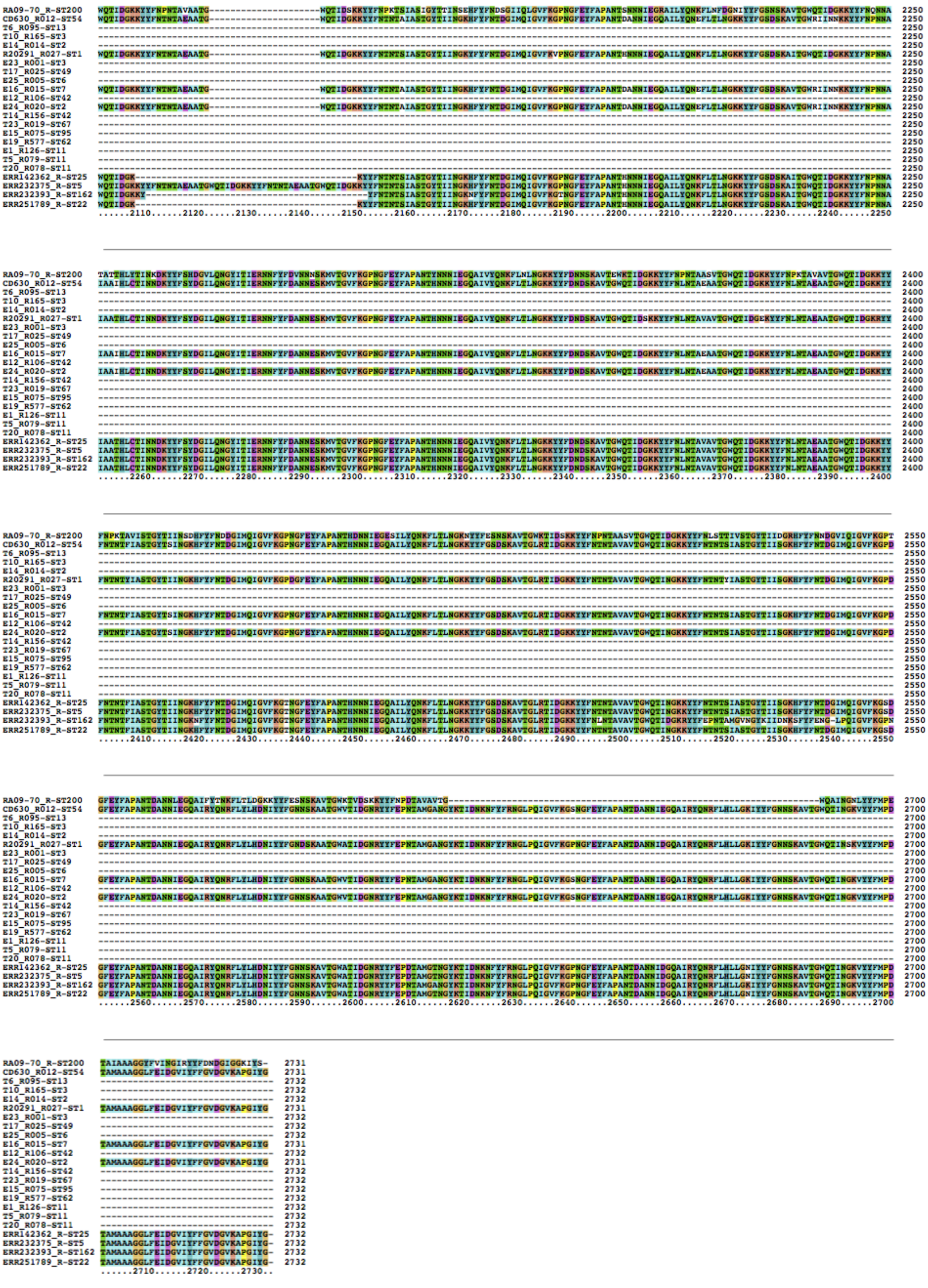

<u>Figure S9.</u> Analysis of TcdB (cf. Material and Methods)

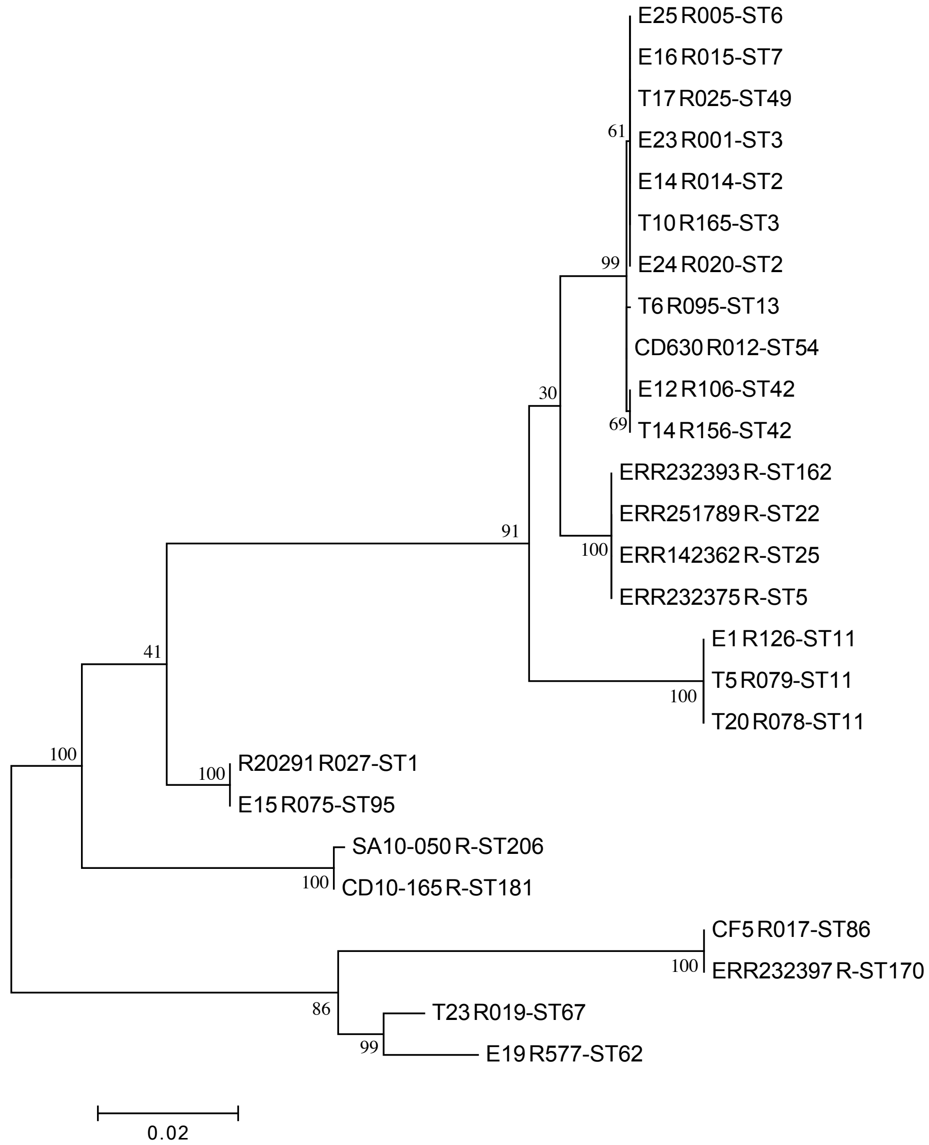

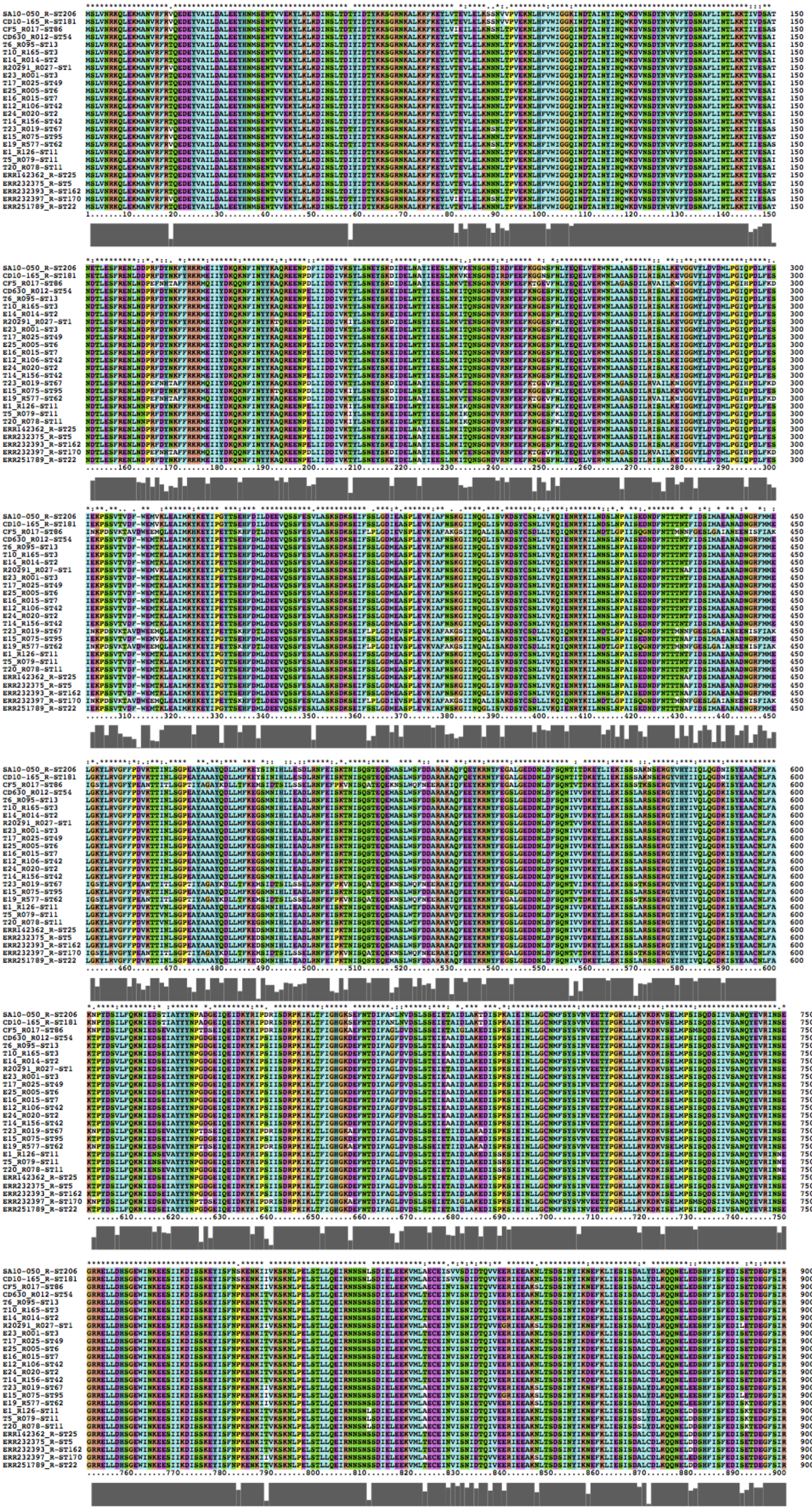
SA10-050_R-ST206
CD10-165_R-ST181
CF5_R017-ST86
CD630_R012-ST54
T6_R095-ST13
T10_R165-ST3
E14_R014-ST2
R20291_R027-ST1
E23_R001-ST3
T17_R025-ST49
E25_R005-ST6
E16_R015-ST7
E12_R106-ST42
E24_R020-ST2
T14_R156-ST42
T23_R019-ST67
E15_R075-ST95
E19_R577-ST62
E1_R126-ST11
T5_R079-ST11
T20_R078-ST11
ERR142362_R-ST25
ERR232375_R-ST5
ERR232393_R-ST162
ERR232397_R-ST170
ERR251789_R-ST22

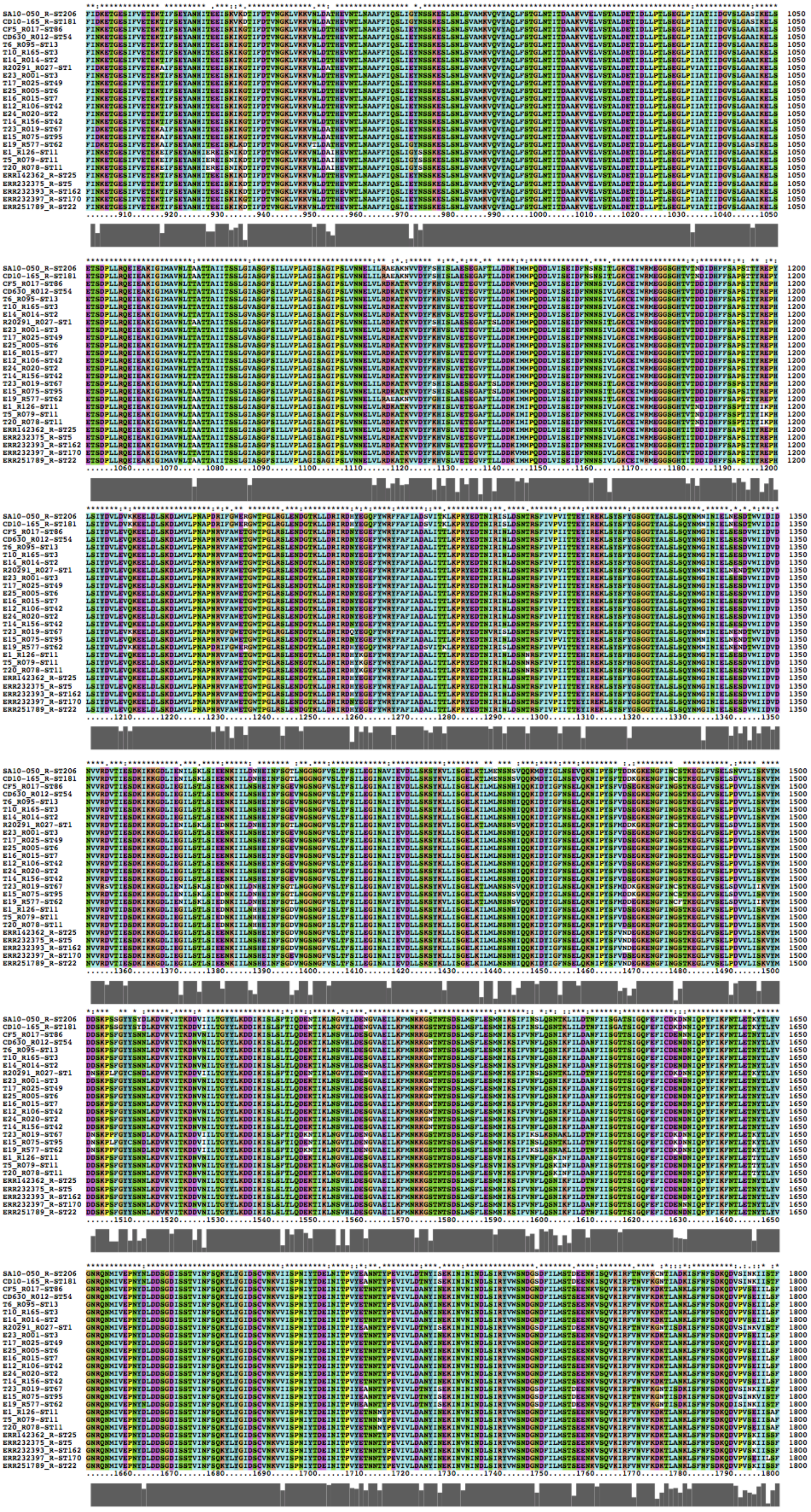

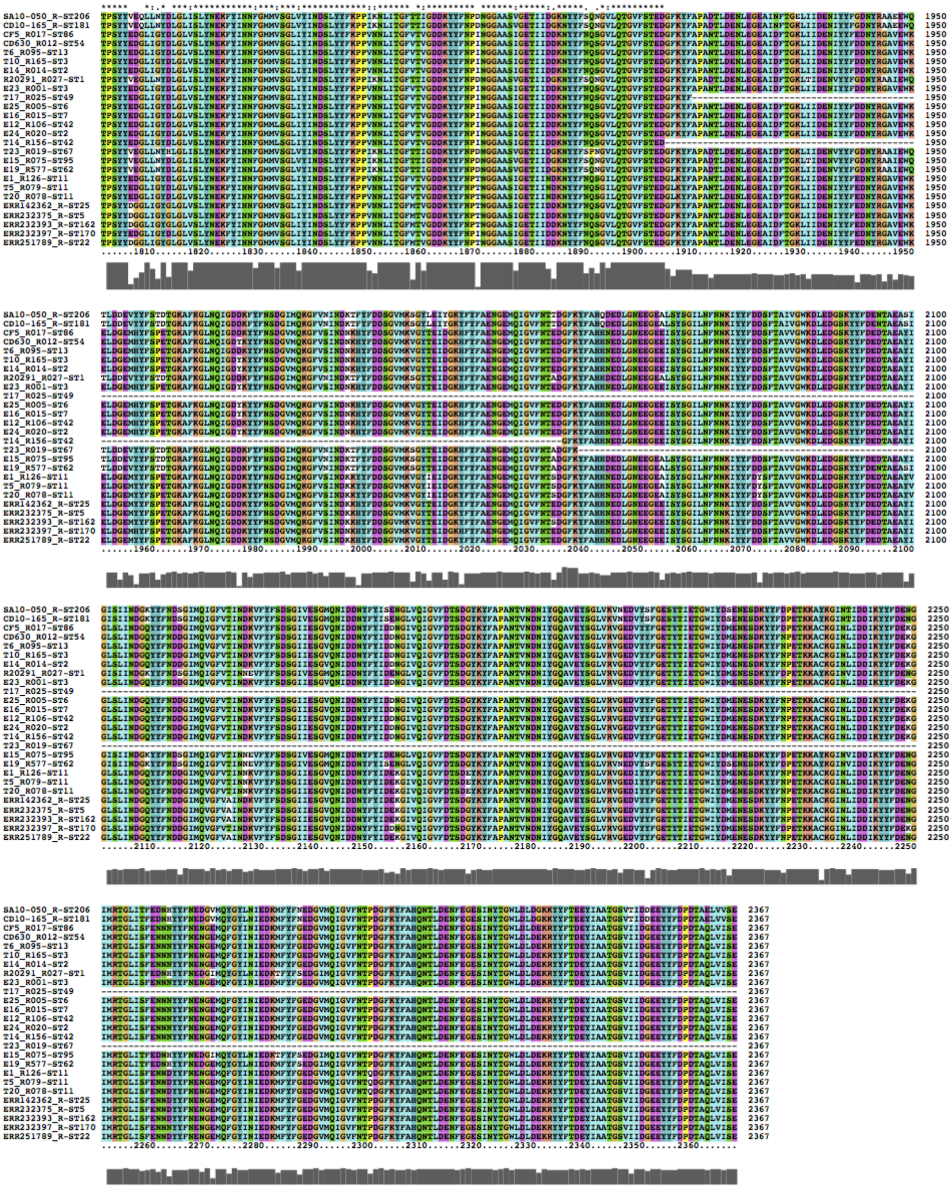

Supplement: Supplementary Information [file srep15023-s1.pdf]
